# A measure of agreement across numerous conditions: assessing when changes in network structures are tissue-specific

**DOI:** 10.1186/s12864-018-5340-3

**Published:** 2019-01-09

**Authors:** Alejandro Cáceres, Juan R. Gonzalez

**Affiliations:** 1 ISGlobal, Doctor Aiguader, Barcelona, 08003 Spain; 2 Centro de Investigación Biomédica en Red en Epidemiología y Salud Pública (CIBERESP), Barcelona, Spain; 3 Department of Mathematics, Universitat Autònoma de Barcelona, Bellaterra, Barcelona, 08193 Spain

**Keywords:** Brain, Transcriptome, Co-expression networks, Reliability, Coehn’s kappa, GTEx, RNA-sequencing, Addiction, Nicotine

## Abstract

**Background:**

There is great interest to study how gene pathways change their structure across different tissues. The assessment of inter-study reliability of pathway changes across tissues can inform on the fraction of tissues with specific functional changes in network structure. However, there is a lack of agreement measures among studies that independently observe how a group of observations change across conditions. We, therefore, propose *λ*, a new inter-study reliability measure that determines the consistency to distinguish observations by condition.

**Results:**

We derived *λ*’s distributional characteristics, determine its reliability properties and compared it with Cohen’s *κ*. We studied the co-expression structure of 287 gene pathways across four brain regions in two transcriptomic studies and applied *λ* to assess the inter-study reliability of the pathways’ brain-regional changes. Brain-related pathways showed highest *λ*; the top value was for the nicotine addiction pathway whose structure was reliably distinguishable among regions with dopaminergic projections.

**Conclusion:**

Our results offer novel substantial evidence that changes in network structure across tissues can be inferred independently of samples, algorithms and experiments (RNA-sequencing or microarrays). Reliability measures, such as *λ*, can inform on the tissues where changes in a network’s structure are likely functional. An R package is available at https://github.com/isglobal-brge/lambda.

**Electronic supplementary material:**

The online version of this article (10.1186/s12864-018-5340-3) contains supplementary material, which is available to authorized users.

## Background

**Fig. 1:**
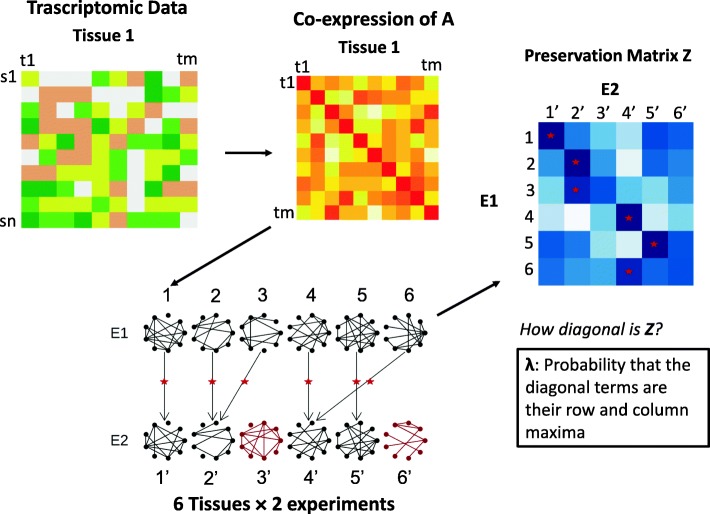
Definition of *λ* for a co-expression network inferred in 6 tissues in two different studies. Schematic representation of a *m*-gene pathway (*A*) whose co-expression structure is inferred from the transcriptomic data of different tissues/conditions (1 to 6) across two studies (E1, E2). The inference for tissue 1 from data is illustrated. The figure shows the preservation matrix *Z* (blue) for the network *A* that is constructed from the pair-wise preservation statistics between tissues across different experiments. The arrows show the pairing between tissues across experiments given by the maximum *Z* found in the rows and columns that cross each diagonal term of *Z*. Network structures in tissues 1, 2, 4 and 5 in E1 are correctly matched by tissue in E2, as their diagonal elements are row and column maxima. Tissues 3 and 6 are not matched correctly. The fraction of tissues for which network structures in E1 can be correctly paired to those in E2 is 4/6, illustrated by the red stars that fall in the diagonal. *λ* is a reliability measure defined as the probability that the diagonal terms in *Z* are their row and column maxima. *λ* informs on how diagonal *Z* is

Reproducibility is a pressing issue in biomedical research that particularly worries a large number of researchers in the field [1]. Research guidelines from leading journals and the American Statistician Association urge for the need of confirmation studies and accurate statistical reporting [2, 3]. In systems biology, gene interaction networks are often derived from the integration of high-throughput data with the aim to determine gene structures with probable biological functionality. Networks, therefore, also need to be reproduced between independent studies in order to contain valid scientific content [4]. Inferred networks or established gene pathways may be additionally assessed under different and numerous conditions to understand the physiological changes of their associated biological functions. The interest here is, for instance, to determine for which tissues a pathway has a specific function [5]. Therefore, if the pathway is physiological only in specific tissues, the inter-study reproducibility of its structure across other tissues, where it is not functional, is expected to be low. As such, neurobiological networks would be expected to meaningfully change in structure between nervous tissues but not between connective tissues like blood [6]. While pair-wise comparisons of a network reproducibility between two studies can be applied on a single tissue [7], there is a need to measure the reproducible changes in network structure across multiple tissues which, in particular, informs on the fraction of tissues for which the changes may underlie tissue-specificity. We, therefore, aimed to propose a measure that allows us to determine the degree to which the changes in network structure among a range of tissues are reproducible across studies; Fig. 1 shows a schematic representation of the data and the type of network reliability we intent to assess.

Classical statistics include numerous ways to measure the reliability of an observation [8]. Reliable observations are reproducible and accurate. Agreement measures between two independent experiments are used to assess the consistency of the observations being made. If observations are categorical, Cohen’s *κ* and its generalizations are typically used [9, 10]; if observations are continuous then a number of correlation measures can be used, such as intra-class correlations [11]. These and other agreement measures are suitable when experiments are performed under comparable, unchanged conditions. When studies are designed to test how a group of individuals change under a range of varying conditions, it is of interest to assess how reliable the changes across conditions are. However, there is a lack of recommended inter-rater measures that assess reliable changes among conditions [8, 12].

A first question that can be asked is the degree to which the most similar observations between studies correspond to those under the same condition, and hence have a measure of how reliably the observations can be distinguished by condition. Similarity can be assessed by any statistic, namely a preservation statistic, that compares two sets of observations at any particular property. A correlation of the observations between two conditions is an example of one preservation statistic. If a preservation statistic for observations between studies is defined [4], we can ask whether the preservation is maximum when the conditions between studies match. The question can be addressed mathematically by computing a preservation matrix, whose elements are the preservation statistics between conditions across studies, and assess the extent to which the diagonal terms are maxima across rows and columns. In particular, a preservation matrix across conditions can be defined for cross-tabulated tables used in classical inter-study reliability studies and, therefore, the ability to correctly pair observations by condition can be studied in those cases. We thus aimed to construct a reliability measure for the correct condition pairing of observations between studies, compare it with Cohen’s *κ* and apply it to assess the inter-study reproducibility of changes in co-expression network structure across tissues.

The GTEx project is an unprecedented effort to study the gene expression in tens of tissues in hundreds of subjects using RNA-sequencing [13]. It is, therefore, a strong candidate for becoming a preferred benchmark for interaction networks across different tissues. As the number of studies with expression data in multiple tissues is expected to increase, agreement measures against GTEx may serve to assess the reproducibility of network changes across tissues [3]. Some studies that measure gene expression in different tissues are publicly available, one of which is the BRAINEAC project [14]. Here, the gene expression using microarray data was measured in hundreds on individuals, free of neurodegenerative disorders at the time of death, in nine different brain tissues, four of which overlap with those in GTEx. Using our agreement measure between the studies, we assessed the fraction of brain regions for which 287 KEGG pathways [15] reliably changed across the cerebellum, frontal cortex, hippocampus and putamen.

## Results

For two studies that measure the same group of units (subjects, co-expression pairs in a network, etc.) across *k* conditions, we propose to construct the *k*×*k* preservation matrix *Z* between conditions. The matrix element $z_{ij'}\phantom {\dot {i}\!}$ (*i*,*j*=1,...*k*) is the correlation (or any preservation statistic) of the units’ observations between condition *i* in the first study and condition *j* in the second study. When the observations are the connections between all node-pairs in a network then $\phantom {\dot {i}\!}z_{ij'}$ may be any normally distributed statistic where pairwise topologies are compared. Figure 1 shows the case in which a co-expression network *A* of *m* gene transctipts is inferred for *n* subjects in 6 tissues (1,...6) in 2 different experiments (E1 and E2). The matrix *Z* is given by all pair-wise comparisons between tissues for the network connections across both experiments. The arrows show the values of *Z* that are maxima across the rows and columns that intersect at each diagonal term.

We are interested in measuring how reproducible the changes of units observations, such as network edges, are. In the case of perfect inter-study reproducibility across all conditions/tissues then the matrix *Z* looks diagonal. That is, $\phantom {\dot {i}\!}z_{ii'}$ is maximum across row *i* and column *i*^′^, for all *i*. In general, this is not the case and the preservation corresponding to row *i* and column *j*^′^≠*i*^′^ may be maximum across the elements in row *i* and column *i*^′^. Figure 1 shows, for instance, that the element of *Z* at tissue 3 in E1 and tissue 2 in E2 is the maximum in row 3 and column 3 of *Z*. As the network structure of tissue 3 in E1 is paired with that of tissue 2 in E2, network connections in tissue 3 have not been reliably reproduced between experiments. By contrast, all the structures at tissues 1, 2, 4 and 5 are correctly paired between experiments, as the corresponding diagonal terms are their row and column maxima and, therefore, their differences are reproduced between experiments.

We define a measure *λ* on the preservation matrix that tells us how diagonal *Z* is. In general, we assume that the units’ observations corresponding to row *i* can be paired by similarity with those in column *j*^′^ if the preservation statistic $\phantom {\dot {i}\!}z_{ij'}$ is maximum across row *i* and column *i*^′^. Note that, as a consequence, *Z* is non-symmetric. We then define *λ* as the expected fraction of correct condition pairings between experiments (*R*), given by

1 $$\begin{array}{*{20}l} \lambda &= E[\!R] = \frac{1}{k} \sum_{i} p_{ii}  \end{array} $$

2 $$\begin{array}{*{20}l} \sigma^{2} &= Var[\!R] = \frac{1}{k^{2}} \sum_{i} p_{ii}(1-p_{ii}) \end{array} $$

where *p*_*ii*_ is the probability that the diagonal term $z_{ii'}\phantom {\dot {i}\!}$ is maximum within its row and column elements. *λ* measures the fraction of conditions that can be reliably distinguished by structural changes, taking into account a probability for random pairing. We modeled *p*_*ii*_ assuming independence among the similarities of observations between conditions, and normality in the preservation statistic (see “Materials and methods” section). As an agreement measure, we derived its reliability properties and compare it with a classical measure of inter-study agreement, Cohen’s *κ*. We then applied it to determine which of 287 KEGG pathways presented higher rates of correct condition pairing across four brain regions between two independent transcriptomic studies.

### Comparison between *λ* and Cohen’s *κ* in simulation studies

We studied the properties of *λ* with extensive simulations. While *λ* is applicable to more general situations than those covered by Cohen’s *κ*, we compared the performance of *λ* with *κ* in simulated cases where both measures can be used. We simulated numerous agreement tables between studies, in which 500 units were classified by both studies into numerous categories/conditions, under different scenarios. From the cross-tabulation table of agreement, see expression 14 in “Materials and methods” section, we computed *κ*: the inter-study reliability of classification that accounts for random agreement. From the corresponding preservation matrix obtained from Eqs. 17 and 18 in “Materials and methods” section, we computed *λ*: the fraction of conditions that can be correctly distinguished between experiments. We also computed *P*_0_: the proportion of units correctly classified between experiments; and *r*: the observed fraction of conditions that are correctly paired between experiments.

**Fig. 2:**
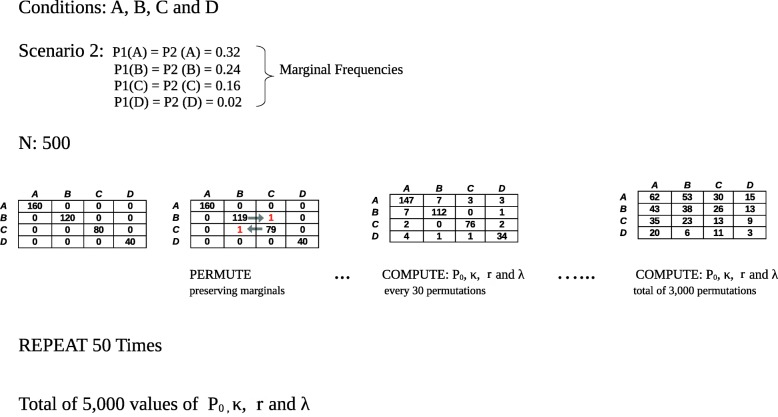
Simulation scheme for reliability measures under one of the proposed scenarios (scenario 2). In the figure, four conditions/tissues are considered (*A*,*B*,*C*,*D*) in which two raters (1,2) measure 500 subjects. The cross-tabulation tables between raters are shown, all with marginal frequencies across raters given by ∼ 1/*j* (*j*=1,2,3,4 and *A*=1,*B*=2,*C*=3,*D*=4), corresponding to the simulation scenario number 2 described in “Materials and methods” section. Initially, all 500 individuals are correctly classified between raters. 3000 permutations are performed, such that the marginals frequencies are conserved. Every 30 permutations reliability measures (*P*_0_, *κ*, *λ* and *r*) are computed. The process is repeated 50 times. A second simulation is performed from an initial cross-tabulation of perfect disagreement, where diagonal terms are all 0. A total of 10,000 values of each measure are then obtained for simulations that cover the whole reliability interval (0,1) under each scenario

We recreated the reproducibility assessment between two raters/experiments across varying number of conditions/tissues *k*=(5,7,15), where three types of cross-tabulation tables between raters across conditions were allowed (expression 14 in “Materials and methods” section). Those were tables with: 1) marginal equiprobabilities 1/*k* of both experiments across all tissues (scenario 1), 2) marginal probabilities ∼ 1/*j* for tissue *j* (scenario 2) and 3) marginal probabilities ∼ 1/*j*^2^ (scenario 3), where *j*=1,...*k*. Simulation scenarios 2 and 3 were designed to test situations where agreement between studies tend to concentrate around one tissue *j*=1. We performed 10,000 simulations per number of conditions and scenario. Simulations were extracted from the permutation of 50 diagonal cross-tabulation tables (perfect agreement) and 50 cross-tabulation tables with null diagonals (perfect disagreement). Conditions across subjects were permuted a total of 30,000 for each table, preserving their marginal frequencies, given by each simulated scenario. Every 30 permutations, agreement measures were computed obtaining a total of 5000 values for perfect agreement (Fig. 2) and 5000 for perfect disagreement. Simulations thus covered the entire interval between perfect agreement (*P*_0_=1) and null agreement (*P*_0_=0). See Additional file 1: Table S1 and “Materials and methods” section for further details.

Simulations showed that when null agreement is expected (*P*_0_=0) *λ* is 0, while *λ* is 1 for full agreement (*P*_0_=1). Consistently with this, we observed that *λ* increased monotonically with *κ* for all the simulation scenarios, see Additional file 2: Figure S1. The functional dependence was highly stable under different scenarios, revealing, as expected, high *λ* agreement for fair values of *κ* (0.2,0.4), given that the latter is a measure of exact agreement rather than discriminative agreement. Therefore, the agreement measured by *λ* is consistently higher than the agreement measured by *κ*. We also observed that for low values, *λ* tends to zero when *κ* takes small negative values, a situation already described in Cohen’s work [9]. For a given *κ*, we observed a sizable range of *λ* values, in particular, as tissues become less equiprobable (scenario 3). We noted that if the number of tissues is small (*k*=5) and the marginal distribution greatly concentrates around one single tissue (*j*=1 for scenario 3), then *λ* tends to 1/*k* (0.2) because the experiments can clearly distinguish that tissue from the rest. In this case, *κ* tends to zero.

**Fig. 3:**
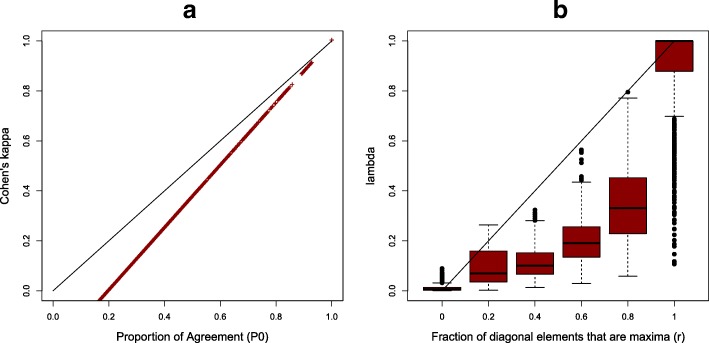
Comparison between agreement measures that correct for random agreement with those that do not. **a** Cohen’s *κ* is compared with *P*_0_ (the total fraction of agreement). **b** *λ* is compared with *r* (the observed fraction of diagonal elements that in the preservation matrix are their row and column maxima)

We then studied the relationship between the agreement measures that account for random agreement with those that do not. In our simulations, we confirmed that *κ* is lower than the proportion of agreement *P*_0_ (Fig. 3); which illustrates the initial motivation for *κ*’s definition as a measure that corrects for random agreement [9]. Similarly, *r*, the observed fraction of times the diagonal terms in the preservation matrix are row and column maxima (Eq. 20 “Materials and methods” section), is higher than *λ*, a distributional estimate of such fraction. Note also that *r*, as a realization of the random variable *R*, is discontinuous with *k*+1 possible values, while *λ* is a continuous variable ranging from 0 to 1.

We additionally studied the variance *σ* of the fraction *R*, for which *r* is a realization and *λ* its expected value. We observed that *σ* decreased with the number of tissues and with departure from marginal equiprobability (Additional file 3: Figure S2). We observed that for a given *λ*, multiple values of *σ* are allowed (Additional file 4: Figure S3). However, the mean of *κ*, for a single draw of the binomial process in Eq. (16), is a function of its variance. In particular, *κ*’s variance is minimum at extreme values (0,1) and is inversely proportional to the number of subjects [16]. In contrast, we observed that *σ* decreases towards zero for a range of values of *λ*. This occurs when *λ* tends to *r*, that is, when the probabilities that the diagonal terms of the preservation matrix are maxima tend either to zero or to one, see Eq. (13) in “Materials and methods” section. We also computed the variability of *λ* from the 5% confidence intervals (CI) obtained from bootstrapping the units observations in the cross-tabulation tables. For simulations corresponding to 5 equiprobable conditions and varying number of subjects, we observed that the length of *λ*’s CI was proportional to *σ* and independent of the number of subjects (Additional file 5: Figure S4).

Overall, these observations show that the fraction of tissues for which network changes are reproducible can be determined with high precision, low *σ* and therefore small CIs for *λ*. From a practical point of view, low or moderate values of *λ* can be used to select the tissues for which the network changes can be reliably measured, and are likely tissue-specific. Consider, for instance, a situation where the structure of a gene pathway is inferred in 50 different tissues. If we expect that the network is biologically functional in only 5 of the tissues, then *λ* will be at most 0.1. In addition, low *σ* will also indicate that the network structure in 90% of tissues could be discarded on grounds of measurement reliability. By contrast, *κ* is maximally informative only as it tends to 1, that is when all the conditions are distinguishable and the preservation matrix is fully diagonal.

We finally performed a power test for *λ*, where we simulated a true agreement scenario in which 3/5 tissues could be reliably paired across two studies. We simulated a true scenario using a multivariate distribution for 10 random variables corresponding to 5 networks per experiment; 3 networks between experiments correlated with strength of 0.5 while all other possible network correlations, across tissues and studies, varied from 0 to 0.5. We also varied the number of genes *N* in the network (10, 20, 40) corresponding to *N*(*N*−1)/2 non-redundant connections (45, 190, 780). We observed that *λ* had sufficient power (> 80*%*) to detect true agreement when the difference between matching tissue correlations and background correlations is 0.05 for 40 gene networks or 0.2 for networks consisting of 10 genes (Additional file 6: Figure S5).

### KEGG pathways

The Kyoto encyclopedia of genes and genomes (KEGG) offers a list of experimentally characterized biochemichal pathways. We computed *λ* between two independent transcriptomic studies, GTEx (RNA-sequencing) and BRAINEAC (microarray), that measured gene expression levels with different technologies in the cerebellum, frontal cortex, hippocampus and putamen.

For each of the 287 KEGG pathways, we selected the commonly annotated genes in each study. We computed the co-expression networks, where genes were hubs and the correlations between the expression levels were edges between hubs. We then derived the pathways’ adjacency matrices from the absolute value of the gene-pair correlations in each tissue. As a pairwise preservation statistic, we used the adjacency correlation (*cor*.*cor*) that quantifies how similar the connectivity is between two network structures [4], see “Materials and methods” section. We then computed the preservation matrices of network connectivities for each pathway. As a result, we obtained a *λ* per pathway. Table 1 shows the pathways with the top *λ* values (> 0.5). While no pathway correctly matched its structure between studies across all four tissues, we observed seven pathways (2%) with agreement between (0.5, 0.75); those are pathways that correctly paired their structures across two to three tissues between studies. Remarkably, five of these pathways are directly linked with signaling processes specific to brain, suggesting that the differences between network structures across brain regions may be tissue-specific. We further observed that in the complete list of all KEGG pathways (Additional file 8: Table S2), brain pathways were highly ranked by *λ*. In the top-ranked pathways, we also observed MicroRNAs in cancer and adrenergic signaling in cardiomyocytes. While these are not brain-specific pathways, numerous MicroRNAs have been shown to express in brain [17] and adrenergic signaling plays an important role in long-term potentiation in hippocampus [18]. We also computed the bootstrap 5% CI of *λ* and estimated *σ* for each pathway. We observed a linear relationship between the length of the interval and *σ*, confirming our previous observation (Additional file 5: Figure S4) that the variability of *λ* is a function of *σ* and independent of the number of genes in the pathways; see Additional file 7: Figure S6).

**Table 1:** Agreement measure *λ*>0.5 between BRAINEAC and GTEx across 4 brain regions in 287 KEGG pathways

*λ*	CI: 2.5%	CI: 97.5%	*σ*	Ref	Description
0.68	0.72	0.75	0.02	hsa05033	Nicotine addiction
0.67	0.61	0.88	0.04	hsa04720	Long-term potentiation
0.58	0.61	0.77	0.04	hsa05206	MicroRNAs in cancer
0.55	0.50	0.59	0.01	hsa04080	Neuroactive ligand-receptor interaction
0.53	0.42	0.69	0.03	hsa04020	Calcium signaling pathway
0.52	0.45	0.65	0.03	hsa04261	Adrenergic signaling in cardiomyocytes
0.51	0.49	0.62	0.02	hsa04912	GnRH signaling pathway

**Fig. 4:**
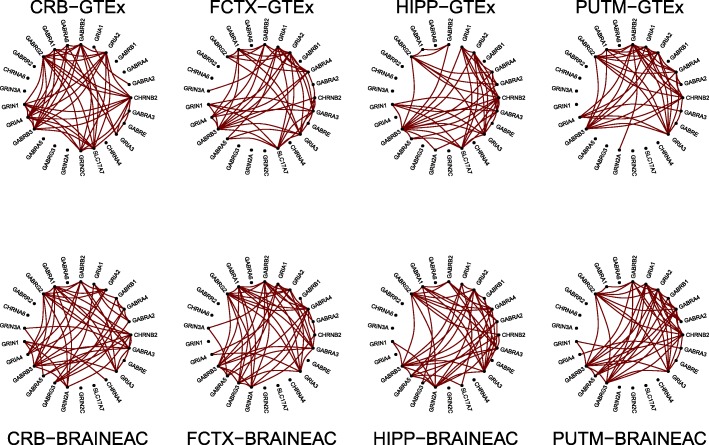
Co-expression structure of the nicotine addiction pathway in the cerebellum (CRB), frontal cortex (FCTX), hippocampus (HIPP) and putamen (PUTM) across the GTEx and BRAINEAC studies. The figure illustrates the strongest correlations between genes only (greater than the last decile)

**Fig. 5:**
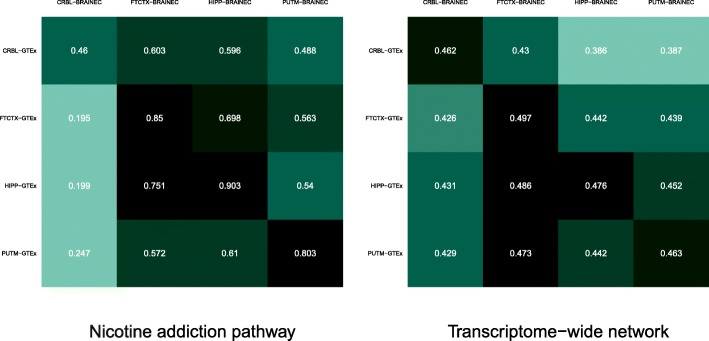
Preservation matrices for the nicotine addiction pathway and the transcriptome-wide network. The elements in the preservation matrices are Fisher’s Z-transformed correlations of the co-expression relationships among genes between studies

Figure 4 illustrates the structure of the strongest edges, across tissues and studies, of the nicotine addiction pathway, which was the top hit with *λ*=0.67 and *σ*^2^=0.02. We observed that the structure of the network was preserved between studies in which, for instance, *GABRB3* was an important hub across tissues and studies while some genes like *GABRR2* and *CHRNA6* were consistently unlinked from the network. While some reliable differences between tissues can be noticed, the preservation matrix in Fig. 5 clearly shows that the frontal cortex, the hippocampus and the putamen can be correctly paired between studies, while the cerebellum cannot. The lower value of *λ*=0.67 from the observed *R*=0.75 is due to the variability of the preservation values and, perhaps, to the closeness in structure between the hippocampus and the frontal cortex.

We also computed benchmark- *λ* as a measure of benchmarking the pathway’s structures obtained from BRAINEAC against GTEx, (Table 2 and Additional file 8: Table S3). Benchmark- *λ* corresponded to the expected value of tissues from BRAINEAC that were correctly paired to those in GTEx, given by the maximum over rows of the preservation matrix at a given column (see “Materials and methods” section). We observed higher values of benchmark- *λ*, and in particular, 20 pathways with benchmark- *λ*>0.7, 15 of which were brain-related. Seven of those pathways are involved in synaptic signaling (glutamatergic, cholinergic, GABAergic and dopaminergic synapse, and long-term potentiation, retrograde endocannabinoid and calcium signaling pathways), and four in addiction processes (nicotine, morphine, amphetamine and alcohol).

**Table 2:** Agreement measure *λ*>0.7 for the benchmarking of BRAINEAC against GTEx across 4 brain regions in 287 KEGG pathways

*λ*	CI: 2.5%	CI: 97.5%	*σ*	Ref	Description
0.97	0.99	1.00	0.01	hsa04724	Glutamatergic synapse
0.95	0.97	1.00	0.01	hsa05033	Nicotine addiction
0.94	0.93	1.00	0.01	hsa04360	Axon guidance
0.91	0.93	1.00	0.02	hsa05414	Dilated cardiomyopathy
0.89	0.97	1.00	0.02	hsa04931	Insulin resistance
0.84	0.88	0.99	0.03	hsa04725	Cholinergic synapse
0.84	0.82	0.97	0.02	hsa05032	Morphine addiction
0.82	0.78	0.93	0.02	hsa04720	Long-term potentiation
0.82	0.76	0.89	0.02	hsa05031	Amphetamine addiction
0.81	0.76	0.95	0.02	hsa04912	GnRH signaling pathway
0.79	0.70	0.91	0.03	hsa04261	Adrenergic signaling in cardiomyocytes
0.76	0.75	0.78	0.01	hsa04727	GABAergic synapse
0.75	0.75	0.75	0.00	hsa04080	Neuroactive ligand-receptor interaction
0.75	0.75	0.75	0.00	hsa04723	Retrograde endocannabinoid signaling
0.74	0.75	0.75	0.00	hsa04020	Calcium signaling pathway
0.74	0.73	0.76	0.01	hsa04728	Dopaminergic synapse
0.74	0.76	0.90	0.03	hsa05206	MicroRNAs in cancer
0.73	0.75	0.76	0.01	hsa04015	Rap1 signaling pathway
0.71	0.67	0.95	0.04	hsa04971	Gastric acid secretion
0.71	0.68	0.80	0.02	hsa05034	Alcoholism

### Transcriptome-wide gene network

We inferred the transcriptome-wide co-expression network for 9071 genes across the GTEx and BRAINEAC studies in the four brain tissues. The network was fully characterized by 8.2×10^7^ gene-pair correlations. We observed *λ*=0.5 for the transcriptome-wide network across all four tissues. Figure 5 illustrates the preservation matrix between studies across tissues. We observed that all the preservation statistics in the matrix were similar in size, between 0.38 and 0.49, and that the cerebellum and frontal cortex diagonals were correctly paired between studies. We noted that the diagonal terms at the hippocampus and putamen were the second maxima after their correlations with the frontal cortex in GTEx. Therefore, the transcriptome-wide networks cannot clearly disentangle the frontal cortex from the hippocampus and putamen, suggesting that a large amount of the 8.2×10^7^ gene-pair correlations may not be brain-region specific.

We finally benchmarked the transcriptome-wide network obtained in BRAINEAC against GTEx across the four brain regions. In this case, we confirmed that all diagonal terms were their column maxima (see Fig. 5) and therefore benchmark- *λ*=1. These results show that the transcriptome-wide network can correctly pair BRAINEAC tissues with those of GTEx. As the benchmarking only takes into account the variability between tissues of GTEx and not BRAINEAC, the observation: benchmark- *λ*=1, is consistent with the consideration that RNA-sequencing (GTEx) may provide better characterization of the transcriptome than microarray technology (BRAINEAC) [19].

## Discussion

We proposed a new measure, *λ*, of agreement between studies. The motivation of the measure is the assessment of agreement between studies that measure the effects of varying conditions on a set of units (subjects, co-expression pairs, etc). We showed that the measure conformed to the properties of agreement measures and used simulations to compare it with Cohen’s *κ*. Our results illustrate the large potential of *λ*, in particular, for studying the changes in gene-network structure across numerous tissues. The measure is particularly useful to determine the fraction of tissues for which network structures can be reliably distinguished, and thus informs on the ratio of structural changes that may be tissue-specific.

In our application to co-expression networks in brain, we observed that the structure of a number of brain-related pathways could be reliably matched by tissue across GTEx and BRAINEAC. We observed that the nicotine addiction pathway presented the highest *λ* in which the frontal cortex, the hippocampus and the putamen were reliably distinguished. Interestingly the cerebellum, with a limited role in addiction, could not be reliably distinguished. Benchmarking of BRAINEAC against GTEx, confirmed that the differences in the nicotine pathway structure across relevant brain regions were highly reliable, as were those in glutamatergic, cholinergic, GABAergic, dopaminergic synapse pathways, whose complex interactions are critical to nicotine addiction [20]. The reliability of other addiction pathways (morphine, amphetamine and alcohol) supports these findings and indicates that structural changes of those pathways in regions with dopamine projections, which are essential in addiction processes (frontal cortex, hippocampus and putamen), are likely tissue-specific [21].

The agreement measure *λ* is based on the preservation matrix between tissues. Langfelder and colleagues thoroughly studied a number of pair-wise preservation statistics of Cholesterol Biosynthetic Process network across sex and tissue differences in mice [4]. Their approach to assessing the reproducibility of the data was to test the preservation of network structure against a reference set of conditions (female/liver), allowing them to identify the condition (male/liver) for which structures are most similar. However, their data show, more generally, that sex changes are much smaller than tissue changes and, therefore, that differences in network structures can reliably pair tissues between sex changes. In this case, the inter-study reliability for changes in the network structure across a range of tissues is clearly high, as it would be summarized by a by high *λ*. While a pair-wise preservation statistic assesses the reliability of a network’s structure between two conditions [4], the preservation matrix registers all pair-wise changes of the structure among numerous conditions. *λ* is a measure on the preservation matrix that allows the assessment of agreement between studies to reliably discriminate between conditions, measuring the probability that the diagonal terms of the matrix are their row and column maxima.

We are unaware of similar measures of agreement on akin preservation matrices that test the changes in the structure of a network across tissues. Other functions of the preservation matrix could also be investigated. We observed, in particular, that *λ* is a measure of inter-observer agreement that is consistently higher than Cohen’s *κ*. Perfect agreement for *κ* is exclusively given by diagonal tables, while perfect agreement for *λ* is given by maxima diagonal terms in tables where their elements, irrespective of their magnitude, are estimated with sufficient low variability. *λ* does not test the level of pair-wise preservation but the extent to which structural patterns are correctly classified by conditions. This is an important difference between the measures, which allows *λ* to be used in more general situations where the elements of cross-tabulated tables between raters/studies are inferences. In particular, we observed that *λ* is informative for intermediate values, as it can be used to select the fraction of tissues where network changes are reproducible and likely to be tissue-specific.

Currently, the validity of a gene or protein network derived from high-throughput data is often benchmarked against networks derived from current knowledge of specific interactions, given by curated pathways, specific experiments or even text mining of published articles [22]. This type of confirmatory analysis extracts networks that are a mixture of interactions that have been individually reported on different tissues. Therefore, while validity is investigated, in terms of consistency with previous knowledge, reproducibility of network structure in an independent confirmatory study is not being assessed. We observed important preservation of network patterns between two equivalent, independent studies, based on different technologies (RNA-seq and microarray), analysis methods and subjects. As such, our results fully test inter-observer reproducibility and provide support that inferred network structures are biological entities independent of numerous sources of variability and heterogeneity in the studies. Previous work has tested the agreement of transcription measurements between RNA-sequencing and microarray experiments on the same subject samples ([23, 24]). These are important studies to validate experimental techniques. However, fully testing inter-observer reproducibility of network inferences, as we have done, must be based on independent confirmatory experiments on different population samples, experiments and analyses. Finally, recent efforts have been made to assess Bayesian network reproducibility, where intra-study preservation statistics have been proposed [25]. While there is still a need to apply those metrics to assess inter-study reproducibility, note that *λ* will also be applicable in such a case.

## Conclusions

We propose a novel inter-study measure of agreement *λ* to determine the fraction of conditions for which structural changes in a set of observations may be deemed reliable. The measure revealed that changes in the co-expression structure of addiction-related pathways can correctly distinguish the frontal cortex, hippocampus and putamen but not cerebellum between studies. Reliable tissue differences in network structure can help to identify tissue-specific pathway-biology and increase the reproducibility of network inferences. More generally, our agreement measure can be used in a set of independent studies that measure how the same group of units changes across numerous conditions.

## Materials and methods

We propose an inter-study measure of agreement to discriminate conditions. While the measure can be applied to different research studies, such as those with factorial designs, we illustrate how its need arises from an example in current functional genomic research. We consider two studies that measure the same units (subjects, gene-pair correlations, etc.) across multiple conditions. We aim to construct a measure that informs on the fraction of conditions that can be reliably distinguished by structural changes in the observations.

### The problem

Let us assume that we have two studies that measure the expression of a set of genes in two different population samples, in the same range of tissues and using different experimental setups. For instance, one experiment may use RNA-seq and the other a microarray technology. We may be interested in inferring the co-expression relationships between the genes across tissues and determine whether the changes in the relationships are consistent between experiments. The co-expression between two genes in the network is given by their correlation over the subjects’ gene expression levels. Figure 1 illustrates the situation where the co-expression between the genes of a network is inferred for 6 tissues in two different studies. Our aim is then to propose a measure that tells us the fraction of tissues that can be reliably distinguished by the network structure across studies. The measure will then inform on the fraction of tissues for which the changes in gene relationships may be tissue-specific.

A network *A* of *m* genes (nodes) can be represented by a *m*×*m* adjacency matrix between genes, where the entries are the edges linking a pair of nodes, see Fig. 1. We are interested in gene-network modules, such as pathways or gene-sets associated with a biological function. There are numerous structural properties of networks that can be used to test the preservation of a network between two studies. Preservation statistics quantify given aspects of within-network topology that are preserved between studies, see Langfelder et al. for a review [4]. Given a preservation statistics *z*, we aim to assess the inter-study reliability of a network’s structure between studies *E*1 and *E*2 across numerous conditions.

For studies that compute a network across different conditions like tissues (*i*=1,...*k*), we want to assess the preservation between studies of the network changes across tissues. We thus form the preservation matrix *Z* across conditions where the entries are pair-wise preservation statistics $z\left (A_{i},A'_{j}\right)=z_{ij'}\phantom {\dot {i}\!}$ of network *A* between tissues *i* and *j* in *E*1 and *E*2, respectively. For three tissues we thus have

3 $$ Z = \left(\begin{array}{lll} z_{11'} & z_{12'} & z_{13'} \\ z_{21'} & z_{22'} & z_{23'} \\ z_{31'} & z_{32'} & z_{33'} \end{array} \right)   $$

We would then like to have a measure of inter-study agreement from *Z*, which can tell how diagonal *Z* is or, more explicitly, the extent to which *i* can be correctly paired with *i*^′^, for all *i*.

### A solution

In *Z*, we can pair by similarity two network structures corresponding to condition *i* in *E*1 and condition *j* in *E*2 if the element $\phantom {\dot {i}\!}z_{ij'}$ is maximum within row *i* and column *j*. As such, we are interested in measuring the extent to which two structures can be paired when the conditions match between studies, that is when *j*^′^=*i*^′^. We then propose to measure the probability that the diagonal terms in the preservation matrix are their row and column maxima. For Z in equation 3, we thus compute

- $p_{11}=Pr(z_{11'}> z_{12'}, z_{13'}, z_{21'}, z_{31'}) \phantom {\dot {i}\!}$,
- $p_{22}=Pr(z_{22'}> z_{21'}, z_{23'}, z_{12'}, z_{32'})\phantom {\dot {i}\!}$ and
- $p_{22}=Pr(z_{33'}> z_{31'}, z_{32'}, z_{13'}, z_{23'})\phantom {\dot {i}\!}$,

where *p*_*ii*_ is the probability that the network structures in tissue *i* between studies are correctly paired by maximum similarity. Taken the preservation statistic as a similarity measure, we can assume that the similarity of one structure (*i*) to another (*i*^′^) is independent of its similarity to a third (*j*^′^). Therefore, the probabilities *p*_*ii*_ can be computed as the product of the individual pair-wise probabilities

4 $$ p_{ii}=\prod\limits_{j=1}^{k} Pr(z_{ii'}>z_{ij'}) * Pr(z_{ii'}>z_{ji'}),   $$

where the first factor is the probability that $\phantom {\dot {i}\!}z_{ii'}$ is the maximum over row *i* of *Z*, and the the second factor is the probability that $z_{ii'}\phantom {\dot {i}\!}$ is the maximum over column *i*^′^. We can write down an explicit form for the pair-wise probabilities, further assuming that the preservation statistic distributes normally

5 $$ z_{ij'} \sim N\left(\mu_{ij'},\sigma^{2}_{ij'}\right).  $$

Following the independence of structural similarities between experiments in different tissues, we then have that the pair-wise probability $\phantom {\dot {i}\!}Pr(z_{ii'}>z_{ij'})$ can be derived from the suitable integration of the joint distribution of $\phantom {\dot {i}\!}z_{ii'}$ and $\phantom {\dot {i}\!}z_{ij'}$

6 $$\begin{array}{*{20}l} Pr(z_{ii'}>z_{ij'})&\,=\, \int_{-\infty}^{\infty} \int_{z_{ij'}}^{\infty} N\left(\mu_{ii},\sigma_{ii}^{2}\right) N\left(\mu_{ij'},\sigma_{ij'}^{2}\right) dz_{ii'}dz_{ij'}. \end{array} $$

7 $$\begin{array}{*{20}l} &=\frac{1}{2} \left[1- \text{erf}\left(\frac{1}{\sqrt{2}}\frac{\mu_{ij'} -\mu_{ii}}{\sqrt{\sigma_{ij'}^{2} +\sigma_{ii}^{2}}}\right)\right].  \end{array} $$

where erf is the error function [26]. Therefore, we have that the probability that the diagonal term $\phantom {\dot {i}\!}z_{ii'}$ is the maximum in the row *i* is

8 $$ \prod\limits_{j=1}^{k} Pr(z_{ii'}>z_{ij'}) = \frac{1}{2} \prod\limits_{j=1}^{k} \left(1- \text{erf} \left[ \frac{1}{\sqrt{2}}\frac{\mu_{ij'} -\mu_{ii}}{\sqrt{\sigma_{ij'}^{2} +\sigma_{ii}^{2}}} \right] \right).  $$

The probability that the diagonal term $z_{ii'}\phantom {\dot {i}\!}$ is the maximum in the column *i* follows a similar form.

Our agreement measure then follows from the fraction *R* of diagonal terms in *Z* that are their row and column maxima. The fraction *R* distributes as the fraction of successes in *k* Bernoulli trials each of which has its own probability *p*_*ii*_; that is, as a Poisson binomial distribution for the success fraction with mean and variance

9 $$\begin{array}{*{20}l} \lambda &= E[\!R] = \frac{1}{k} \sum_{i} p_{ii}  \end{array} $$

10 $$\begin{array}{*{20}l} \sigma^{2} &= Var[\!R] = \frac{1}{k^{2}} \sum_{i} p_{ii}(1-p_{ii}). \end{array} $$

The agreement measure *λ* is then the expected fraction of conditions with the correct matching of network structures between studies. In the case that *E*1 is the benchmark for experiment *E*2, then one is interested in testing whether the diagonal terms are the maxima of their columns only, generalizing the concepts of sensitivity and specificity. In this case *λ* can be computed by simply setting $Pr(z_{ii}>z_{ji'})=1\phantom {\dot {i}\!}$ for *j*=1,...*k*. Note also that it is straightforward to generalize the measure for more than two studies by expanding the products in Eq. (4).

### Reliability properties of *λ*

Suitable reliability measures satisfy three basic properties: i) their values range from 0 to 1; ii) they tend to 0 when no agreement is expected and tend to 1 when a full agreement is expected and iii) they account for random agreement.

Regarding property i), *λ* clearly ranges from 0 to 1, as it is given by the fraction between the sum of *k* probabilities *p*_*ii*_ and *k*. To show properties ii) and iii), we look at the explicit model for *p*_*ii*_ given by the substitution of Eq. (7) in (4)

11 $$ {\begin{aligned} p_{ii}= \prod\limits_{j=1}^{k} \frac{1}{2} \left[ 1- \text{erf} \left(\frac{1}{\sqrt{2}}\frac{\mu_{ij} -\mu_{ii}}{\sqrt{\sigma_{ij}^{2} +\sigma_{ii}^{2}}} \right) \right] * \frac{1}{2} \left[1- \text{erf} \left(\frac{1}{\sqrt{2}}\frac{\mu_{ji} -\mu_{ii}}{\sqrt{\sigma_{ji}^{2} +\sigma_{ii}^{2}}} \right)\right].  \end{aligned}}  $$

erf(*x*) is the error function which, importantly, defines the Heaviside step function in the limit: $H(x)= 1/2*{\lim }_{t\to 0} \text{erf} (-x/t)$ [26]. Therefore, when {*σ*_*ii*_,*σ*_*ji*_,*σ*_*ij*_}→0, *p*_*ii*_ tends to

12 $$\begin{array}{*{20}l} p_{ii}&= \prod_{j=1}^{k} H(\mu_{ii}>\mu_{ij})*H(\mu_{ii}>\mu_{ji}) \end{array} $$

13 $$\begin{array}{*{20}l} &=\left\{\begin{array}{ll} 1, & \text{if}\ \mu_{ii}> \{\mu_{ij},\mu_{ji}\} \forall j=1...k\\ 0, & \text{otherwise.}  \end{array}\right. \end{array} $$

That is, when there is no variability in *z*_*ij*_, *p*_*ii*_ is 1 if $\phantom {\dot {i}\!}z_{ii'}$ is maximum across rows and columns and 0 otherwise. Therefore, regarding property ii), null and perfect reliabilities are clearly expected with null variability. For such cases all *p*_*ii*_ are either 0 or 1 according to Eq. (13). In the case of null reliability none of the *μ*_*ii*_ is maximum, all *p*_*ii*_ are 0 and *λ*=0. Whereas, in the case of perfect reliability all *μ*_*ii*_ are maxima, all *p*_*ii*_ are 1 and *λ*=1. Regarding property iii), we can see that under null variability, *λ* tends to the *observed* fraction of conditions *r* that are correctly paired between experiments. The fraction *r*, which is a realization of *R*, can be considered as a reliability measure but does not account for random agreement. By contrast, *λ*, as an expected value of *R* under a probability distribution, incorporates a variability that accounts for correct random pairing.

### Comparison between measures of agreement

Let us take two raters who observe *l* units in *k* categories. Then, for instance, if *k*=3 and categories are labeled *a*, *b* and *c*, the cross-tabulation matrix of observaions takes a similar form of expression (3), 
14

where *n*_*i*,*j*_ is the number of units observed in category *i* and *j* by the first and second rater respectively, and ${\sum \nolimits }_{i,j=1}^{k} n_{ij}=l$. Agreement is typically measured by Cohen’s *κ*

15 $$ \kappa= \frac{{\sum\nolimits}_{i=1}^{k}P(i,i) - {\sum\nolimits}_{i=1}^{k}P_{1}(i)P_{2}(i)}{1- {\sum\nolimits}_{i=1}^{k}P_{1}(i)P_{2}(i)},  $$

where *P*(*i*,*i*)=*n*_*ii*_/*l* is the observed frequency of units that were measured in category *i* by both raters and *P*_*d*_(*i*) is the frequency of units in *i* observed by rater *d* (*d*=1,2). The sum $P_{0}= \sum _{i} P(i,i)$ is the total fraction of agreement: the proportion of observations that falls in the diagonal, which does not account for random agreement. Cohen’s *κ* measures the fraction of discordant observations expected by chance that are actually observed in agreement.

While *λ* can be applied to a wider range of reliability studies than *κ*, we compared the two measures in cases where both of them can be computed. Note that a matrix *Z* in Eq. (3) can be computed from the cross-tabulation table in expression (14). Given that in row *i*, in expression (14), the number of observed units is *n*_*i*_=*P*_1_(*i*)∗*l*, we can then assume that *n*_*ij*_ is one draw of a binomial process

16 $$ n_{ij}\sim Binomial(n_{i}, \theta_{ij})   $$

where $\hat {\theta }_{ij}=n_{ij}/n_{i}$, and the mean and variance of the mean are given by

17 $$\begin{array}{*{20}l} \mu_{ij}& = n_{ij}  \end{array} $$

18 $$\begin{array}{*{20}l} \sigma_{ij}^{2}& = n_{ij} (1-n_{ij}/n_{i}),  \end{array} $$

For for large *n*_*i*_ the binomial distribution tends to a normal distribution. Therefore, the values in Eqs. 17 and 18 can be used in Eq. 7. With a similar computation for the column elements, the measure *λ* can be obtained for a table in the form (14) and compared with the value of *κ* for varying values of the total fraction of agreement *P*_0_.

As *κ* is an agreement measure that corrects *P*_0_ for random agreement, we compared *λ* with the *observed* fraction of diagonal elements that are their row and column maxima *r*, explicitly given by

19 $$\begin{array}{*{20}l} r_{i} & =\left\{ \begin{array}{ll} 1, & \text{if} \; n_{ii}=max\left(\left\{n_{ij}, n_{ji}\right\}_{j}\right)\\ 0, & \text{otherwise} \end{array}\right. \end{array} $$

20 $$\begin{array}{*{20}l} r & = \frac{1}{k} \sum_{i} r_{i}.  \end{array} $$

### Simulations

We performed a series of simulations to study the properties of *λ* with respect to *κ* and *r*. Simulations were obtained for three total number of conditions/tissues *k*=(5,7,15), and three fifferent forms for the marginal frequencies *P*_1_(*i*) and *P*_2_(*i*) (*i*=1,...*k*) across studies 1 and 2

- Scenario 1 (equiprobable): $P_{1}(i)=P_{2}(i)=\frac {1}{k}$, ∀*i*
- Scenario 2: $P_{1}(i)=P_{2}(i)=\frac {1}{i} / {\sum \nolimits }_{j=1}^{k} \frac {1}{j} $
- Scenario 3 (the least equiprobable): $P_{1}(i)=P_{2}(i)=\frac {1}{i^{2}} / {\sum \nolimits }_{j=1}^{k} \frac {1}{j^{2}} $

We set the number of observations to *l*=500. For each scenario, we simulated 50 cases of perfect agreement tables (*P*_0_=1), i.e. diagonal matrices, and 50 cases of perfect disagreement (*P*_0_=0); those are tables with zeros on the diagonal terms except for the cell of maximum joint probability. For each case, we permuted a pair of observations 3000 times, such that the original marginal frequencies were conserved. After each set of 30 permutations, we computed the four agreement measures. This procedure allowed the assessment of 10,000 simulations, in each scenario and tissue level, covering the whole agreement interval of *P*_0_. The simulation scheme is shown in Fig. 2 and further details are given in Additional file 1: Table S1.

We used R.3.30 and the package psych to perform calculations and compute Cohen’s *κ*.

### Gene expression data

We downloaded expression data from the GTEx project obtained from RNA-seq [27]. Reads per kilobase per million mapped reads (RPKM) of version 6 were obtained for all brain tissues. Covariates for each tissue were also downloaded.

We also downloaded the brain expression data of the BRAINEAC project [28] obtained from winsorized values of exon array data (Affymetrix Human Exon 1.0 ST array). Downloaded data had been previously normalized and corrected for batch effects.

We identified four brain tissues common in both data-sets and for which GTEx had covariates’ information. Those were cerebellum (CRBL) with 125 individuals in GTEx and 130 in BRAINEAC, frontal cortex (FCTX) with 108 and 135 individuals, (HIPP) hippocampus with 94 and 130 individuals, and putamen (PUTM) with 82 and 135 individuals, respectively.

Between the two studies, we mapped 9071 common genes to compute all gene-pair correlations of expression levels.

### Co-expression networks

For each tissue in GTEx, we downloaded its corresponding table of covariates. These included 20 variables, 3 of which are genome-wide principal components that inform on ancestry, 15 surrogate transcriptomic variables that inform on batch effects, one variable containing gender and another platform. As expression levels for GTEx are derived from count data, we performed Spearman’s partial correlations of the expression levels between each gene-pair, among 9071 genes. We used the par.cor function of the ppcor R package to test the rank correlations adjusting for covariates. A transcriptome-wide adjacency matrix was constructed for each tissue, where the entries, corresponding to the edges of the genome-wide network, were the absolute value of all gene-pair correlations.

For the BRAINEAC data, similar adjacency matrices per tissue were obtained. In this case, however, the downloaded gene expression values had already been normalized and corrected for batch effects. As gene expression levels are close to normality, we performed a Pearson’s correlation between all gene-pairs and used these values to compute the adjacency matrices.

We computed *λ* to test the inter-study agreement of the co-expression networks of 287 pathways, downloaded from https://www.genome.jp/kegg/. For each pathway, we extracted a total of eight adjacency matrices (4 tissues × 2 studies) whose elements were the absolute value of the expression correlations between all gene pairs within the pathway. We computed the pathways’ vectorized adjacency matrices, which are the vectors with non-redundant components

21 $$ \begin{aligned} &vectorizeMatrix(A)\\&=(a_{2,1},a_{3,1},a_{3,2},a_{4,1},a_{4,2},a_{4,3},...,a_{n,1},a_{m,m-1}), \end{aligned}  $$

where *m* in the number of genes in the pathway *A*. As a preservation statistic between studies, we used the adjacency correlation (cor.cor in [4]), based on the Pearson’s correlation between the vectorized matrices of pathway *A*

22 $$ \begin{aligned} &cor.cor\left(A_{i},A'_{j}\right)\\&=cor\left(vectorizeMatrix(A_{i}), vectorizeMatrix\left(A'_{j}\right)\right) \end{aligned}  $$

between tissues *i* and *j*, across experiments E1 and E2, respectively. The elements *z*_*ij*_ of the preservation matrix *Z* for each pathway *A* were obtained by the Fisher’s Z-transformation of *cor*.*cor*(*A*_*i*_,*Aj*′).

## Additional files

Additional file 1 **Table S1.** Simulation details. (52.6 KB)

Additional file 2 **Figure S1.** Relationship between *λ* and Cohen’s *κ* across all simulation scenarios. (406 KB)

Additional file 3 **Figure S2.** Relationship between *λ* and *σ* across all simulated scenarios. (401 KB)

Additional file 4 **Figure S3.** Left) Relationship between *κ* and its variance for the simulated scenario with 5 conditions and a single subject, corresponding to a single draw of the binomial process in Eq. (16). The functional dependence of *κ* variance with *κ* is unchanged but reduced in magnitude with increasing *N*. Right) Relationship between *λ* and *σ* in the same scenario. (120 KB)

Additional file 5 **Figure S4.** Bootstrap 5% confidence intervals for *λ* for a simulation with 5 conditions and a varying number of individuals. We see that the length of the CI is proportional to *σ* and independent of the number of subjects. (7.61 KB)

Additional file 6 **Figure S5.** Power estimations for true agreement *r*=3/5 when the true correlations between matching tissues (diagonal terms in the preservation matrix) are fixed at *cor*=0.5 and all other background correlations *c*0, given by all other elements in the preservation matrix, varied from 0 to 0.5. The figure shows the differences between fixed and varying correlations (*corDiff*=*cor*−*cor*0) by a different number of genes *N* in the network. Networks were simulated as random variables from multivariate distributions. (4.90 KB)

Additional file 7 **Figure S6.** Bootstrap 5% confidence intervals for *λ*, between GTEx and BRAINEAC, for 287 KEGG pathways. We see that the length of the CI is proportional to *σ*, regardless of the different number of genes of each pathway. (13.4 KB)

Additional file 8 **Tables S2-S3.** Agreement measure *λ* between GTEx and BRAINEAC for 287 KEGG pathways across four brain regions. (56.0 KB)

Additional file 9 Computer Code. Entire computer code in R to reproduce the results discussed in the manuscript. (12.1 KB)

## Abbreviations

*B**i**n**o**m**i**a**l*(*n*,*p*): Binomial distribution of *n* trials with success probability *p*
BRAINEAC study: The brain eQTL almanac study
CRBL: Cerebellum
erf(*x*): Error function of *x*
FCTX: Frontal cortex
GTEx study: Genotype-tissue expression study
HIPP: Hippocampus
KEGG: The Kyoto encyclopedia of genes and genomes
netA: Network A
PUTM: Putamen
RPKM: Reads per kilobase per million mapped reads
RNA-seq: RNA sequencing
*N*(*μ*,*σ*^2^): Normal distribution with mean *μ* and variance *σ*^2^
