# Supplementary material for: A measure of agreement across numerous conditions: assessing when changes in network structures are tissue-specific
Source: BMC Genomics. 2019 Jan 9;20:26. doi: 10.1186/s12864-018-5340-3 (PMC6327576; doi:10.1186/s12864-018-5340-3)
Supplement: Supplementary file 1 — Table S1. Simulation details. (52.6 KB) [file 12864_2018_5340_MOESM1_ESM.pdf]

Preservation matrices

	Number of conditions		
	5	***10***	***15***
**Scenario 1**	P1(1) ~ 1/5 … P1(5) ~ 1/5	P1(1) ~ 1/10 … P1(5) ~ 1/10	P1(1) ~ 1/15 … P1(5) ~ 1/15
**Scenario 2**	P1(1) ~ 1 …. P1(5) ~ 1/5	P1(1) ~ 1 …. P1(5) ~ 1/10	P1(1) ~ 1 …. P1(5) ~ 1/15
**Scenario 3**	P1(1) ~ 1 …. P1(5) ~ 1/25	P1(1) ~ 1 …. P1(5) ~ 1/100	P1(1) ~ 1 …. P1(5) ~ 1/225

Permutations

	Number of conditions		
	5	***10***	***15***
Scenario 1	subjects=500	subjects=500	subjects=500
	Perm=3,000 from P_0=0	Perm=3,000 from P_0=0	Perm=3,000 from P_0=0
	Perm=3,000 from P_0=1	Perm=3,000 from P_0=1	Perm=3,000 from P_0=1
	Repeat 50 times	Repeat 50 times	Repeat 50 times
	150,000 preservation matrices	150,000 preservation matrices	150,000 preservation matrices
	10,000 values of $P_{0,}$ κ, r and λ	10,000 values of $P_{0,}$ κ, r and λ	10,000 values of $P_{0,}$ κ, r and λ
Scenario 2	subjects=500	subjects=500	subjects=500
	Perm=3,000 from P_0=0	Perm=3,000 from P_0=0	Perm=3,000 from P_0=0
	Perm=3,000 from P_0=1	Perm=3,000 from P_0=1	Perm=3,000 from P_0=1
	Repeat 50 times	Repeat 50 times	Repeat 50 times
	150,000 preservation matrices	150,000 preservation matrices	150,000 preservation matrices
	10,000 values of $P_{0,}$ κ, r and λ	10,000 values of $P_{0,}$ κ, r and λ	10,000 values of $P_{0,}$ κ, r and λ
Scenario 3	subjects=500	subjects=500	subjects=500
	Perm=3,000 from P_0=0	Perm=3,000 from P_0=0	Perm=3,000 from P_0=0
	Perm=3,000 from P_0=1	Perm=3,000 from P_0=1	Perm=3,000 from P_0=1
	Repeat 50 times	Repeat 50 times	Repeat 50 times
	150,000 preservation matrices	150,000 preservation matrices	150,000 preservation matrices
	10,000 values of $P_{0,}$ κ, r and λ	10,000 values of $P_{0,}$ κ, r and λ	10,000 values of $P_{0,}$ κ, r and λ

Table S1. Permutation details. Following the scheme in Figure 2. Preservation tables were defined from the cross-tabulation of subjects assigned to different conditions across two studies. Preservation tables were tested across three different number of conditions (5,10,15) and three scenarios, with different levels of equiprobability for their marginals. 150,000 preservation tables were produced at each combination of schenarios and number of conditions. The tables were produced by starting at tables with perfect reliability (P_0=1, diagonal matrices) and null reliability (P_0=0, null diagonal terms) and permuting labels such that the marginals were conserved. 3,000 permutations were conducted in each case and every 30 permutations the reliability scores were computed. The process was repeated 50 times for each combination of scenarios and number of conditions.
