# Supplementary material for: A measure of agreement across numerous conditions: assessing when changes in network structures are tissue-specific
Source: BMC Genomics. 2019 Jan 9;20:26. doi: 10.1186/s12864-018-5340-3 (PMC6327576; doi:10.1186/s12864-018-5340-3)
Supplement: Supplementary file 3 — Figure S2. Relationship between λ and σ across all simulated scenarios. (401 KB) [file 12864_2018_5340_MOESM3_ESM.pdf]

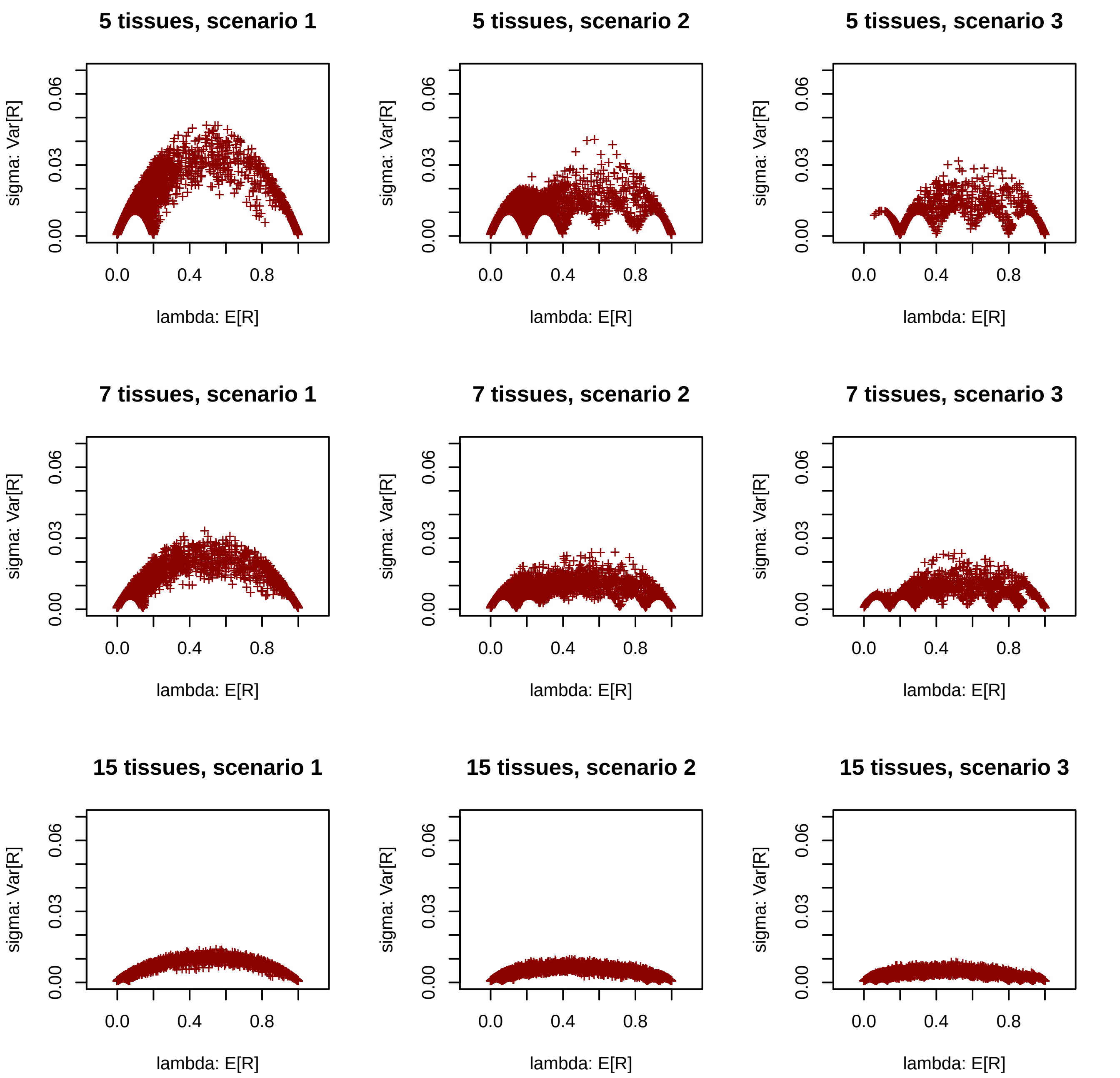
5 tissues, scenario 1
5 tissues, scenario 2
5 tissues, scenario 3
7 tissues, scenario 1
7 tissues, scenario 2
7 tissues, scenario 3
15 tissues, scenario 1
15 tissues, scenario 2
15 tissues, scenario 3
sigma: Var[R]
lambda: E[R]
0.00
0.03
0.06
0.0
0.4
0.8
