# Supplementary figures and images for: A measure of agreement across numerous conditions: assessing when changes in network structures are tissue-specific
Source: BMC Genomics. 2019 Jan 9;20:26. doi: 10.1186/s12864-018-5340-3 (PMC6327576; doi:10.1186/s12864-018-5340-3)

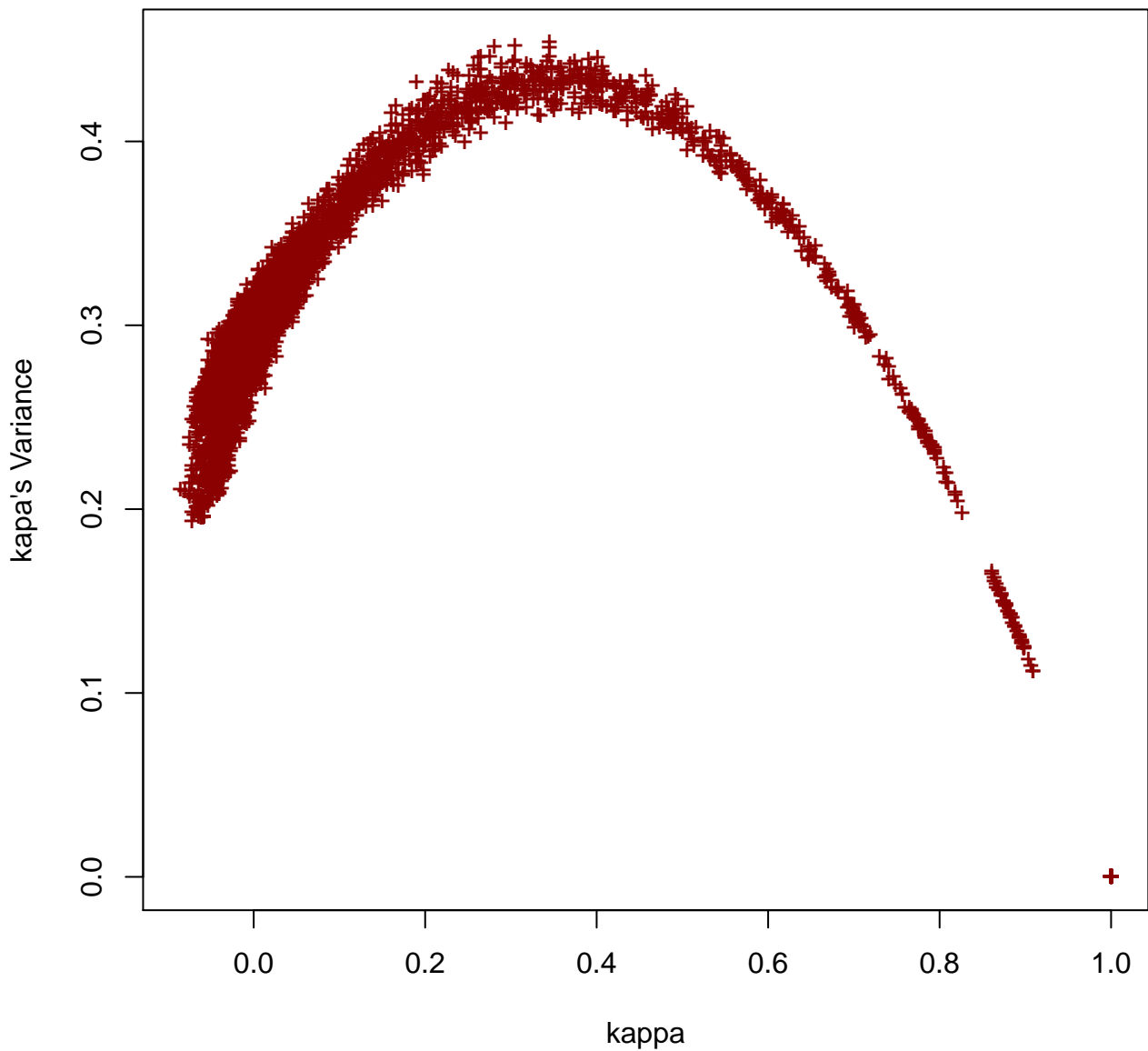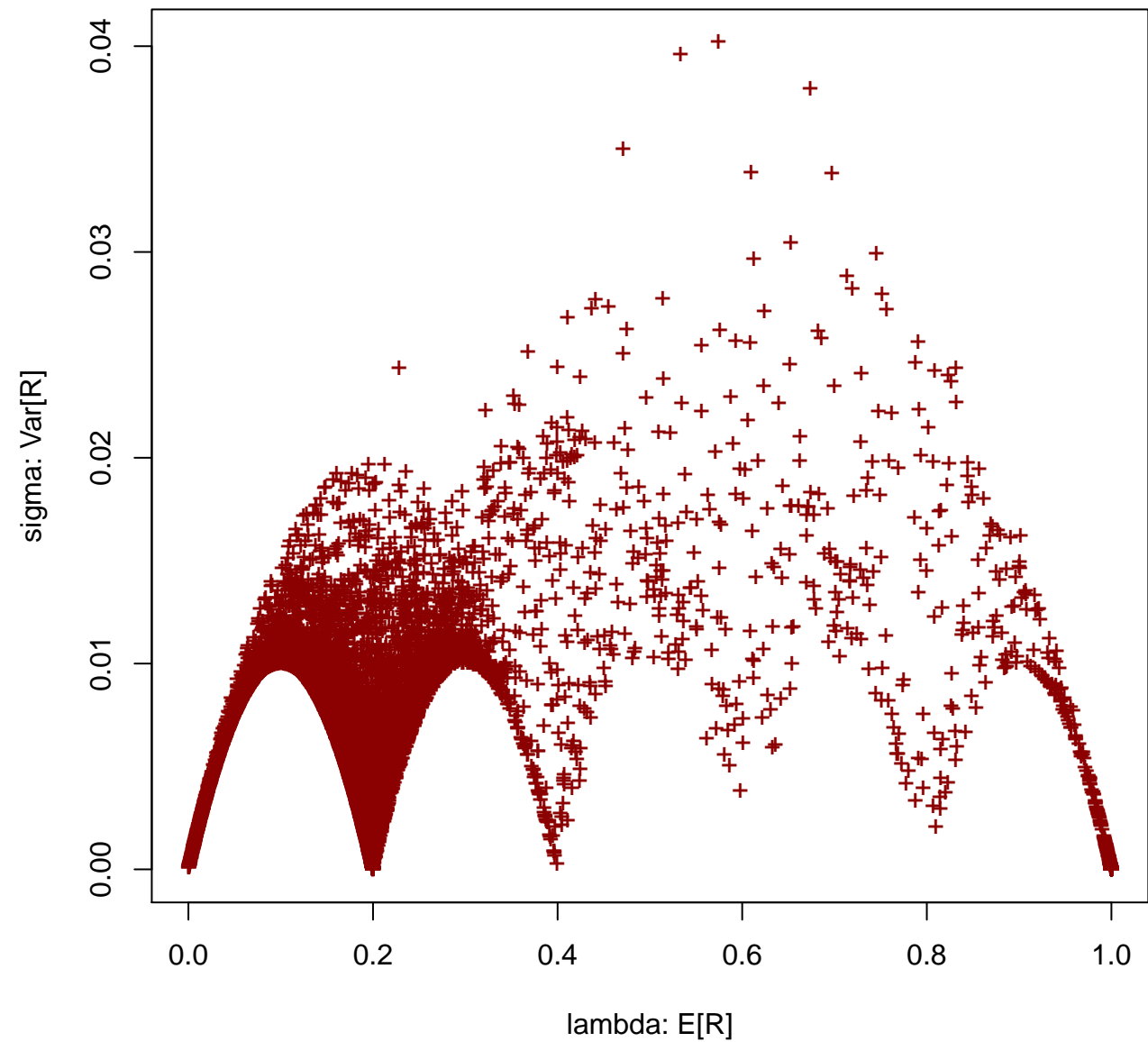

Supplement: Supplementary file 4 — Figure S3. Left) Relationship between κ and its variance for the simulated scenario with 5 conditions and a single subject, corresponding to a single draw of the binomial process in Eq. (16). The functional dependence of κ variance with κ is unchanged but reduced in magnitude with increasing N. Right) Relationship between λ and σ in the same scenario. (120 KB) [file 12864_2018_5340_MOESM4_ESM.pdf]

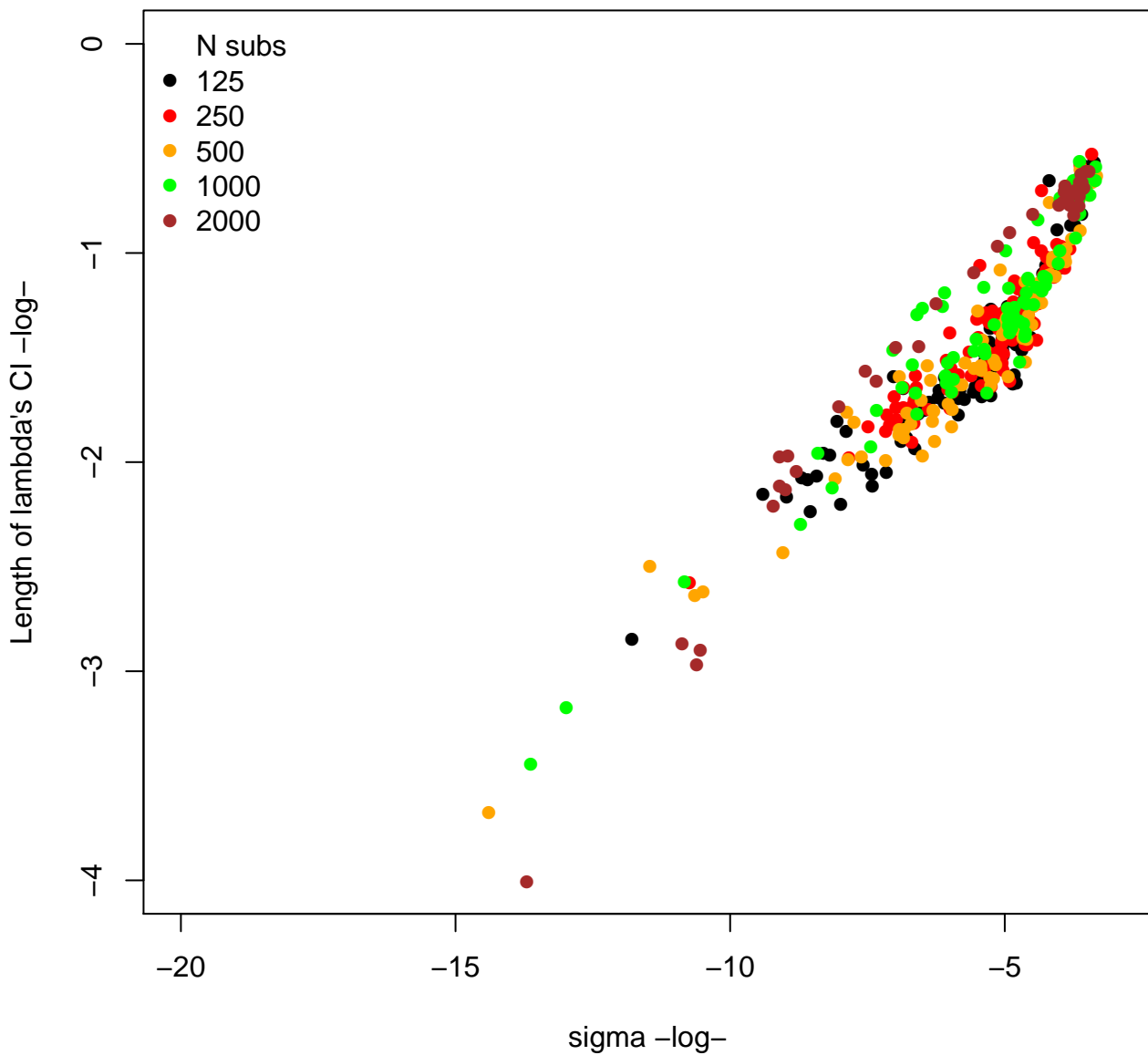

Supplement: Supplementary file 5 — Figure S4. Bootstrap 5% confidence intervals for λ for a simulation with 5 conditions and a varying number of individuals. We see that the length of the CI is proportional to σ and independent of the number of subjects. (7.61 KB) [file 12864_2018_5340_MOESM5_ESM.pdf]

**Power Vs cor Diff by N**  
 **$\alpha=0.05\%$ ,  $\Lambda_0=3/5$ , N tissues=5,  $\text{cor}=0.5$**

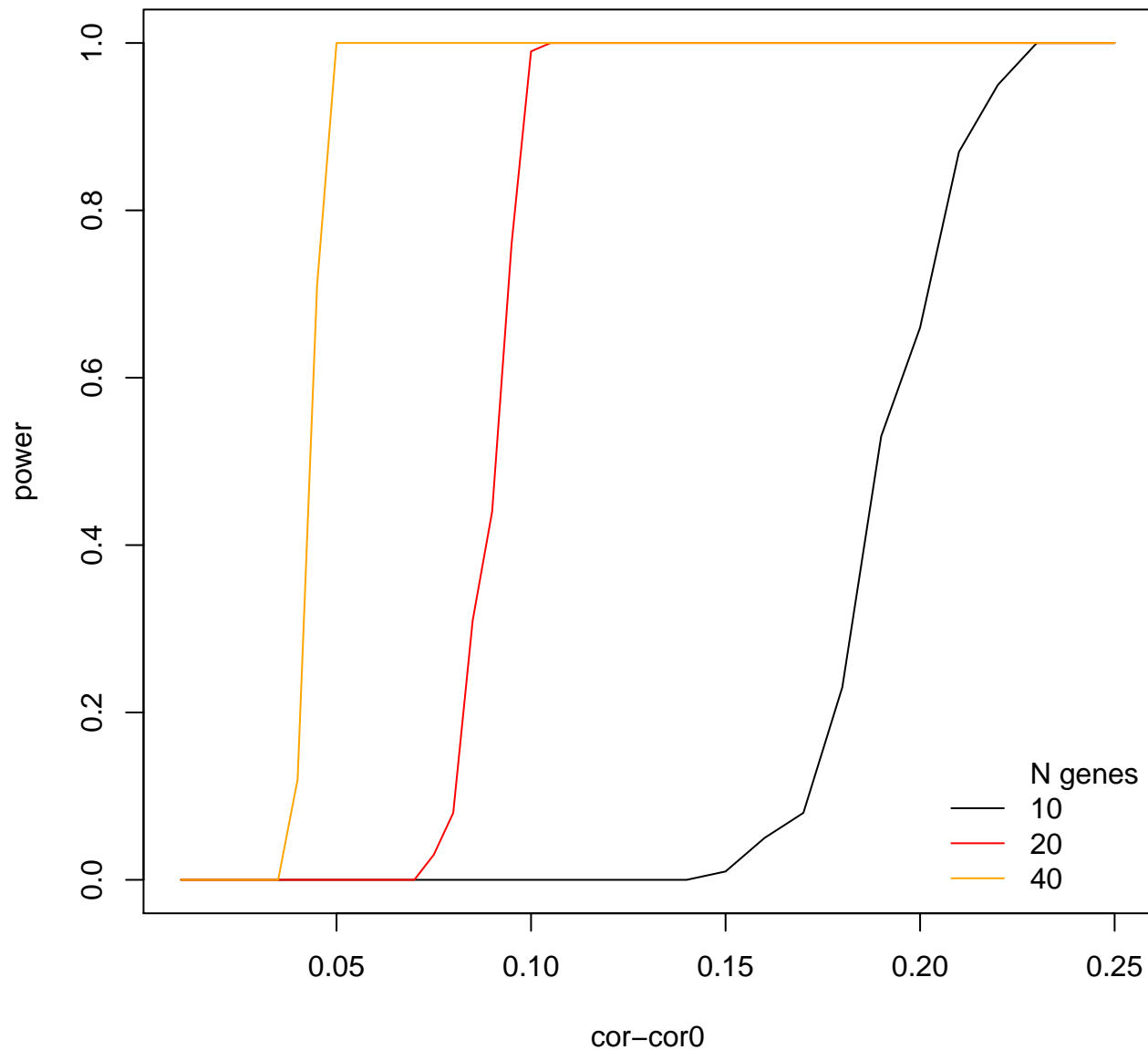

Supplement: Supplementary file 6 — Figure S5. Power estimations for true agreement r=3/5 when the true correlations between matching tissues (diagonal terms in the preservation matrix) are fixed at cor=0.5 and all other background correlations c0, given by all other elements in the preservation matrix, varied from 0 to 0.5. The figure shows the differences between fixed and varying correlations (corDiff=cor−cor0) by a different number of genes N in the network. Networks were simulated as random variables from multivariate distributions. (4.90 KB) [file 12864_2018_5340_MOESM6_ESM.pdf]

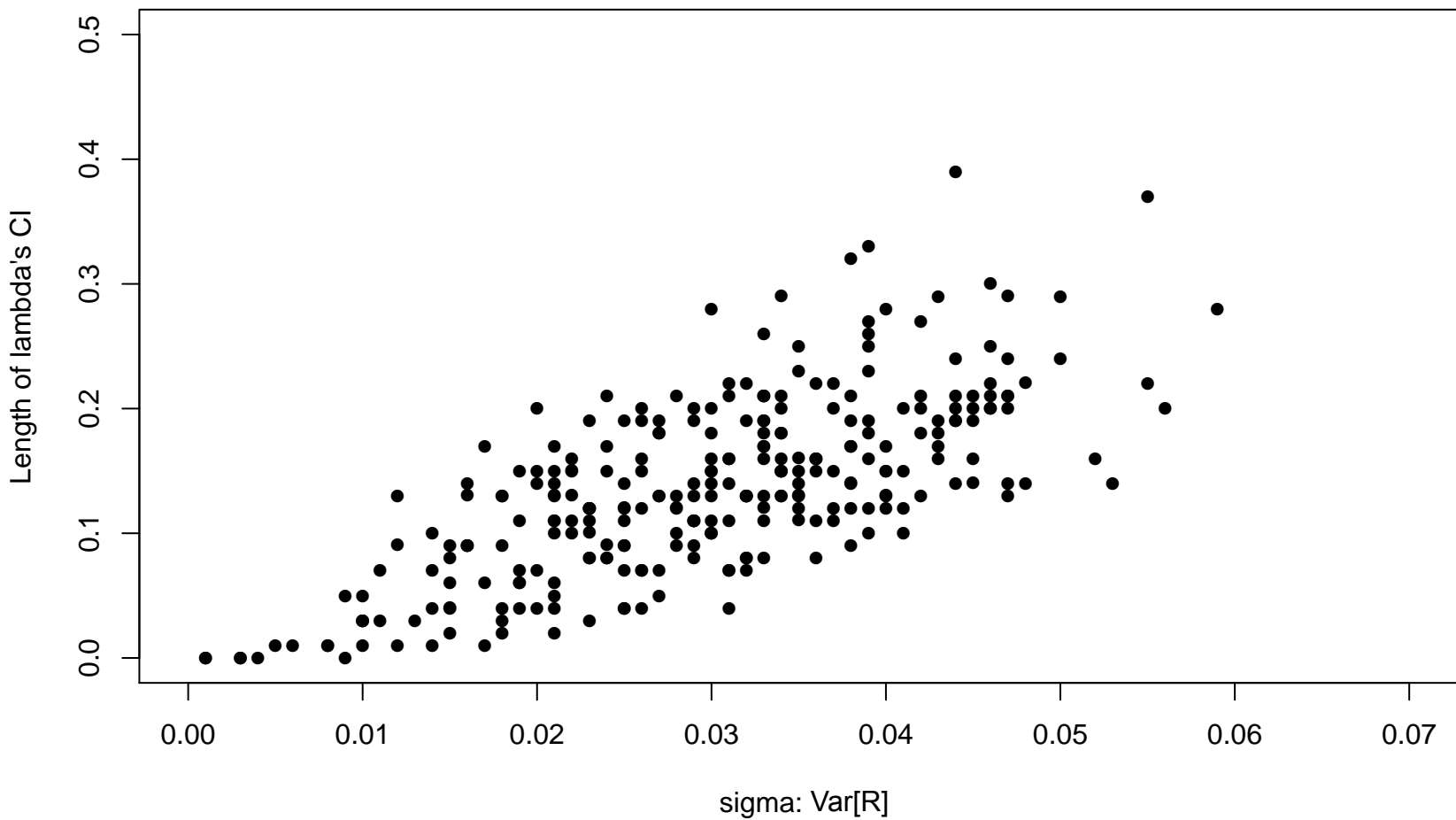

Supplement: Supplementary file 7 — Figure S6. Bootstrap 5% confidence intervals for λ, between GTEx and BRAINEAC, for 287 KEGG pathways. We see that the length of the CI is proportional to σ, regardless of the different number of genes of each pathway. (13.4 KB) [file 12864_2018_5340_MOESM7_ESM.pdf]
