# Supplementary material for: A measure of agreement across numerous conditions: assessing when changes in network structures are tissue-specific
Source: BMC Genomics. 2019 Jan 9;20:26. doi: 10.1186/s12864-018-5340-3 (PMC6327576; doi:10.1186/s12864-018-5340-3)
Supplement: Supplementary file 8 — Tables S2-S3. Agreement measure λ between GTEx and BRAINEAC for 287 KEGG pathways across four brain regions. (56.0 KB) [file 12864_2018_5340_MOESM8_ESM.pdf]

Table S2. Agreement measure λ between BRAINEAC and GTEX for distinguishability of 4 brain regions in 287 KEGG pathways.

λ	CI: 2.5%	CI: 97.5%	σ	Ref	Description
0.68	0.72	0.75	0.02	hsa05033	Nicotine addiction
0.67	0.61	0.88	0.04	hsa04720	Long-term potentiation
0.58	0.61	0.77	0.04	hsa05206	MicroRNAs in cancer
0.55	0.50	0.59	0.01	hsa04080	Neuroactive ligand-receptor interaction
0.53	0.42	0.69	0.03	hsa04020	Calcium signaling pathway
0.52	0.45	0.65	0.03	hsa04261	Adrenergic signaling in cardiomyocytes
0.51	0.49	0.62	0.02	hsa04912	GnRH signaling pathway
0.49	0.47	0.70	0.03	hsa04725	Cholinergic synapse
0.47	0.50	0.64	0.02	hsa03015	mRNA surveillance pathway
0.46	0.37	0.59	0.02	hsa04015	Rap1 signaling pathway
0.46	0.36	0.69	0.05	hsa04713	Circadian entrainment
0.46	0.37	0.54	0.03	hsa05034	Alcoholism
0.46	0.46	0.68	0.04	hsa05145	Toxoplasmosis
0.45	0.42	0.63	0.02	hsa04072	Phospholipase D signaling pathway
0.45	0.54	0.72	0.04	hsa04380	Osteoclast differentiation
0.43	0.43	0.60	0.03	hsa04142	Lysosome
0.43	0.38	0.67	0.06	hsa04540	Gap junction
0.42	0.29	0.51	0.03	hsa05031	Amphetamine addiction
0.42	0.45	0.57	0.03	hsa05164	Influenza A
0.41	0.40	0.64	0.04	hsa04971	Gastric acid secretion
0.41	0.40	0.61	0.03	hsa05169	Epstein-Barr virus infection
0.41	0.44	0.65	0.03	hsa05202	Transcriptional misregulation in cancer
0.40	0.37	0.57	0.02	hsa04114	Oocyte meiosis
0.40	0.43	0.51	0.03	hsa05152	Tuberculosis
0.39	0.35	0.63	0.05	hsa04740	Olfactory transduction
0.39	0.27	0.53	0.04	hsa05414	Dilated cardiomyopathy
0.38	0.34	0.49	0.02	hsa04211	Longevity regulating pathway
0.38	0.30	0.48	0.03	hsa04728	Dopaminergic synapse
0.38	0.29	0.49	0.02	hsa05205	Proteoglycans in cancer
0.37	0.32	0.56	0.04	hsa05203	Viral carcinogenesis
0.36	0.26	0.44	0.02	hsa04360	Axon guidance
0.36	0.34	0.64	0.04	hsa04611	Platelet activation
0.35	0.22	0.51	0.04	hsa04024	cAMP signaling pathway
0.35	0.38	0.63	0.03	hsa04670	Leukocyte transendothelial migration
0.35	0.38	0.66	0.04	hsa04920	Adipocytokine signaling pathway
0.35	0.32	0.47	0.03	hsa05032	Morphine addiction
0.34	0.36	0.49	0.03	hsa03013	RNA transport
0.34	0.30	0.47	0.03	hsa04145	Phagosome
0.34	0.44	0.58	0.03	hsa04664	Fc epsilon RI signaling pathway
0.33	0.36	0.51	0.03	hsa04710	Circadian rhythm
0.33	0.29	0.55	0.04	hsa04742	Taste transduction
0.33	0.28	0.46	0.04	hsa05231	Choline metabolism in cancer
0.31	0.28	0.46	0.04	hsa04144	Endocytosis
0.31	0.28	0.48	0.03	hsa04666	Fc gamma R-mediated phagocytosis
0.31	0.25	0.30	0.02	hsa04727	GABAergic synapse
0.30	0.28	0.40	0.02	hsa04062	Chemokine signaling pathway
0.30	0.25	0.55	0.04	hsa04071	Sphingolipid signaling pathway
0.30	0.26	0.40	0.03	hsa04722	Neurotrophin signaling pathway
0.30	0.25	0.37	0.03	hsa04931	Insulin resistance
0.30	0.25	0.32	0.01	hsa05030	Cocaine addiction

0.29	0.24	0.34	0.02	hsa04724	Glutamatergic synapse
0.28	0.25	0.30	0.01	hsa04151	PI3K-Akt signaling pathway
0.28	0.30	0.48	0.03	hsa05014	Amyotrophic lateral sclerosis (ALS)
0.28	0.22	0.47	0.04	hsa05142	Chagas disease (American trypanosomiasis)
0.27	0.25	0.32	0.01	hsa04120	Ubiquitin mediated proteolysis
0.27	0.25	0.35	0.01	hsa04152	AMPK signaling pathway
0.27	0.25	0.34	0.01	hsa04914	Progesterone-mediated oocyte maturation
0.27	0.14	0.42	0.04	hsa04925	Aldosterone synthesis and secretion
0.26	0.24	0.37	0.02	hsa00270	Cysteine and methionine metabolism
0.26	0.28	0.47	0.03	hsa04146	Peroxisome
0.26	0.18	0.46	0.04	hsa04210	Apoptosis
0.26	0.21	0.43	0.02	hsa04913	Ovarian steroidogenesis
0.25	0.25	0.25	0.00	hsa04150	mTOR signaling pathway
0.25	0.25	0.25	0.00	hsa04213	Longevity regulating pathway - multiple species
0.25	0.29	0.44	0.03	hsa04330	Notch signaling pathway
0.25	0.25	0.28	0.00	hsa04520	Adherens junction
0.25	0.22	0.28	0.01	hsa04723	Retrograde endocannabinoid signaling
0.25	0.25	0.25	0.00	hsa04750	Inflammatory mediator regulation of TRP channels
0.25	0.20	0.46	0.04	hsa04922	Glucagon signaling pathway
0.25	0.25	0.27	0.00	hsa05100	Bacterial invasion of epithelial cells
0.25	0.21	0.44	0.04	hsa05161	Hepatitis B
0.25	0.25	0.25	0.00	hsa05200	Pathways in cancer
0.24	0.25	0.31	0.01	hsa04070	Phosphatidylinositol signaling system
0.24	0.24	0.38	0.02	hsa05110	Vibrio cholerae infection
0.24	0.25	0.41	0.02	hsa05140	Leishmaniasis
0.23	0.24	0.41	0.03	hsa04620	Toll-like receptor signaling pathway
0.23	0.17	0.53	0.04	hsa04730	Long-term depression
0.23	0.25	0.25	0.00	hsa04930	Type II diabetes mellitus
0.23	0.25	0.39	0.03	hsa05130	Pathogenic Escherichia coli infection
0.23	0.25	0.26	0.01	hsa05168	Herpes simplex infection
0.22	0.24	0.39	0.02	hsa00620	Pyruvate metabolism
0.22	0.19	0.36	0.02	hsa02010	ABC transporters
0.22	0.23	0.28	0.01	hsa04010	MAPK signaling pathway
0.22	0.14	0.28	0.02	hsa04022	cGMP-PKG signaling pathway
0.22	0.24	0.43	0.03	hsa04141	Protein processing in endoplasmic reticulum
0.22	0.14	0.38	0.04	hsa04924	Renin secretion
0.22	0.11	0.37	0.04	hsa05410	Hypertrophic cardiomyopathy (HCM)
0.21	0.06	0.32	0.04	hsa04260	Cardiac muscle contraction
0.21	0.22	0.40	0.02	hsa04672	Intestinal immune network for IgA production
0.21	0.15	0.34	0.02	hsa04970	Salivary secretion
0.21	0.21	0.26	0.02	hsa05012	Parkinson's disease
0.20	0.20	0.41	0.03	hsa00240	Pyrimidine metabolism
0.20	0.22	0.35	0.02	hsa00600	Sphingolipid metabolism
0.20	0.13	0.31	0.03	hsa04514	Cell adhesion molecules (CAMs)
0.20	0.17	0.43	0.03	hsa04550	Signaling pathways regulating pluripotency of stem cells
0.20	0.22	0.25	0.01	hsa04932	Non-alcoholic fatty liver disease (NAFLD)
0.20	0.17	0.43	0.04	hsa05120	Epithelial cell signaling in Helicobacter pylori infection
0.19	0.25	0.42	0.03	hsa00410	beta-Alanine metabolism
0.19	0.24	0.42	0.03	hsa03320	PPAR signaling pathway
0.19	0.19	0.35	0.02	hsa04962	Vasopressin-regulated water reabsorption
0.19	0.21	0.40	0.03	hsa05160	Hepatitis C
0.18	0.19	0.40	0.03	hsa00310	Lysine degradation
0.18	0.22	0.55	0.04	hsa00512	Mucin type O-Glycan biosynthesis
0.17	0.16	0.38	0.03	hsa04064	NF-kappa B signaling pathway

0.17	0.16	0.25	0.02	hsa04610	Complement and coagulation cascades
0.17	0.11	0.25	0.02	hsa04923	Regulation of lipolysis in adipocytes
0.17	0.20	0.45	0.03	hsa05131	Shigellosis
0.17	0.23	0.36	0.03	hsa05132	Salmonella infection
0.17	0.06	0.22	0.03	hsa05166	HTLV-I infection
0.17	0.18	0.38	0.03	hsa05219	Bladder cancer
0.16	0.21	0.28	0.02	hsa00100	Steroid biosynthesis
0.16	0.10	0.35	0.03	hsa00564	Glycerophospholipid metabolism
0.16	0.03	0.25	0.03	hsa04911	Insulin secretion
0.16	0.06	0.30	0.03	hsa04972	Pancreatic secretion
0.15	0.15	0.38	0.03	hsa00562	Inositol phosphate metabolism
0.15	0.13	0.29	0.02	hsa00565	Ether lipid metabolism
0.15	0.18	0.25	0.02	hsa04974	Protein digestion and absorption
0.14	0.02	0.21	0.03	hsa00230	Purine metabolism
0.14	0.12	0.27	0.02	hsa00260	Glycine, serine and threonine metabolism
0.14	0.14	0.29	0.02	hsa00280	Valine, leucine and isoleucine degradation
0.14	0.09	0.26	0.02	hsa00510	N-Glycan biosynthesis
0.14	0.19	0.32	0.02	hsa00520	Amino sugar and nucleotide sugar metabolism
0.14	0.14	0.34	0.02	hsa03450	Non-homologous end-joining
0.14	0.15	0.33	0.02	hsa04392	Hippo signaling pathway -multiple species
0.14	0.02	0.20	0.03	hsa04726	Serotonergic synapse
0.14	0.09	0.25	0.02	hsa05211	Renal cell carcinoma
0.14	0.19	0.33	0.02	hsa05222	Small cell lung cancer
0.14	0.11	0.27	0.02	hsa05320	Autoimmune thyroid disease
0.13	0.11	0.25	0.02	hsa00071	Fatty acid degradation
0.13	0.10	0.46	0.03	hsa00910	Nitrogen metabolism
0.13	0.08	0.24	0.02	hsa04512	ECM-receptor interaction
0.13	0.08	0.19	0.02	hsa04917	Prolactin signaling pathway
0.13	0.10	0.29	0.02	hsa04977	Vitamin digestion and absorption
0.13	0.06	0.21	0.02	hsa05010	Alzheimer's disease
0.13	0.04	0.20	0.02	hsa05146	Amoebiasis
0.13	0.10	0.24	0.02	hsa05321	Inflammatory bowel disease (IBD)
0.12	0.13	0.27	0.02	hsa00514	Other types of O-glycan biosynthesis
0.12	0.06	0.28	0.02	hsa00640	Propanoate metabolism
0.12	0.11	0.32	0.02	hsa03460	Fanconi anemia pathway
0.12	0.06	0.25	0.02	hsa04640	Hematopoietic cell lineage
0.12	0.03	0.20	0.02	hsa04916	Melanogenesis
0.12	0.13	0.23	0.02	hsa05340	Primary immunodeficiency
0.12	0.03	0.21	0.02	hsa05412	Arrhythmogenic right ventricular cardiomyopathy (ARVC)
0.11	0.07	0.24	0.02	hsa00380	Tryptophan metabolism
0.11	0.03	0.21	0.02	hsa00604	Glycosphingolipid biosynthesis - ganglio series
0.11	0.02	0.22	0.02	hsa04012	ErbB signaling pathway
0.11	0.10	0.20	0.02	hsa04110	Cell cycle
0.11	0.10	0.26	0.02	hsa04130	SNARE interactions in vesicular transport
0.11	0.11	0.29	0.02	hsa04662	B cell receptor signaling pathway
0.11	0.01	0.18	0.02	hsa04915	Estrogen signaling pathway
0.11	0.02	0.19	0.02	hsa05016	Huntington's disease
0.11	0.07	0.22	0.02	hsa05133	Pertussis
0.10	0.04	0.24	0.02	hsa00630	Glyoxylate and dicarboxylate metabolism
0.10	0.03	0.19	0.02	hsa04921	Oxytocin signaling pathway
0.09	0.02	0.23	0.02	hsa00330	Arginine and proline metabolism
0.09	0.04	0.20	0.02	hsa00480	Glutathione metabolism
0.09	0.07	0.28	0.02	hsa00533	Glycosaminoglycan biosynthesis - keratan sulfate
0.09	0.02	0.21	0.02	hsa00590	Arachidonic acid metabolism

0.09	0.01	0.14	0.02	hsa04068	FoxO signaling pathway
0.09	0.04	0.16	0.02	hsa04340	Hedgehog signaling pathway
0.09	0.05	0.22	0.02	hsa04721	Synaptic vesicle cycle
0.09	0.03	0.17	0.02	hsa04964	Proximal tubule bicarbonate reclamation
0.09	0.04	0.23	0.02	hsa05144	Malaria
0.09	0.05	0.22	0.02	hsa05212	Pancreatic cancer
0.09	0.07	0.21	0.02	hsa05332	Graft-versus-host disease
0.08	0.03	0.22	0.02	hsa00051	Fructose and mannose metabolism
0.08	0.03	0.29	0.02	hsa00062	Fatty acid elongation
0.08	0.07	0.28	0.02	hsa00531	Glycosaminoglycan degradation
0.08	0.08	0.22	0.01	hsa00982	Drug metabolism - cytochrome P450
0.08	0.04	0.19	0.02	hsa03008	Ribosome biogenesis in eukaryotes
0.08	0.05	0.17	0.01	hsa03018	RNA degradation
0.08	0.01	0.10	0.02	hsa03050	Proteasome
0.08	0.01	0.09	0.02	hsa04530	Tight junction
0.08	0.06	0.24	0.02	hsa04614	Renin-angiotensin system
0.08	0.02	0.21	0.02	hsa04650	Natural killer cell mediated cytotoxicity
0.08	0.02	0.13	0.02	hsa04919	Thyroid hormone signaling pathway
0.08	0.04	0.19	0.02	hsa04973	Carbohydrate digestion and absorption
0.08	0.04	0.21	0.02	hsa05416	Viral myocarditis
0.07	0.02	0.18	0.02	hsa00010	Glycolysis / Gluconeogenesis
0.07	0.04	0.24	0.02	hsa00603	Glycosphingolipid biosynthesis - globo series
0.07	0.08	0.22	0.01	hsa01040	Biosynthesis of unsaturated fatty acids
0.07	0.02	0.18	0.01	hsa04612	Antigen processing and presentation
0.07	0.01	0.11	0.01	hsa04933	AGE-RAGE signaling pathway in diabetic complications
0.07	0.08	0.22	0.01	hsa05143	African trypanosomiasis
0.07	0.08	0.21	0.02	hsa05204	Chemical carcinogenesis
0.07	0.03	0.22	0.02	hsa05217	Basal cell carcinoma
0.07	0.01	0.11	0.01	hsa05323	Rheumatoid arthritis
0.06	0.02	0.20	0.01	hsa00561	Glycerolipid metabolism
0.06	0.04	0.18	0.01	hsa00980	Metabolism of xenobiotics by cytochrome P450
0.06	0.03	0.14	0.01	hsa03020	RNA polymerase
0.06	0.05	0.22	0.01	hsa03420	Nucleotide excision repair
0.06	0.03	0.15	0.01	hsa04115	p53 signaling pathway
0.06	0.00	0.12	0.01	hsa04270	Vascular smooth muscle contraction
0.06	0.01	0.11	0.01	hsa04370	VEGF signaling pathway
0.06	0.05	0.18	0.01	hsa04621	NOD-like receptor signaling pathway
0.06	0.00	0.13	0.01	hsa04918	Thyroid hormone synthesis
0.06	0.05	0.20	0.01	hsa04960	Aldosterone-regulated sodium reabsorption
0.06	0.01	0.14	0.01	hsa04978	Mineral absorption
0.06	0.01	0.14	0.01	hsa05214	Glioma
0.05	0.02	0.22	0.01	hsa00030	Pentose phosphate pathway
0.05	0.02	0.11	0.01	hsa00061	Fatty acid biosynthesis
0.05	0.04	0.19	0.01	hsa00130	Ubiquinone and other terpenoid-quinone biosynthesis
0.05	0.02	0.13	0.01	hsa00534	Glycosaminoglycan biosynthesis - heparan sulfate / heparin
0.05	0.03	0.18	0.01	hsa00592	alpha-Linolenic acid metabolism
0.05	0.02	0.17	0.01	hsa00601	Glycosphingolipid biosynthesis - lacto and neolacto series
0.05	0.02	0.21	0.01	hsa00760	Nicotinate and nicotinamide metabolism
0.05	0.01	0.18	0.01	hsa03022	Basal transcription factors
0.05	0.00	0.05	0.01	hsa04060	Cytokine-cytokine receptor interaction
0.05	0.03	0.20	0.01	hsa04140	Regulation of autophagy
0.05	0.03	0.15	0.01	hsa04320	Dorso-ventral axis formation
0.05	0.05	0.18	0.01	hsa04744	Phototransduction
0.05	0.00	0.03	0.01	hsa04910	Insulin signaling pathway

0.05	0.02	0.29	0.01	hsa04950	Maturity onset diabetes of the young
0.05	0.01	0.10	0.01	hsa05223	Non-small cell lung cancer
0.05	0.00	0.05	0.01	hsa05224	Breast cancer
0.05	0.00	0.17	0.01	hsa05330	Allograft rejection
0.04	0.01	0.18	0.01	hsa00053	Ascorbate and aldarate metabolism
0.04	0.04	0.17	0.01	hsa00120	Primary bile acid biosynthesis
0.04	0.04	0.15	0.01	hsa00430	Taurine and hypotaurine metabolism
0.04	0.01	0.11	0.01	hsa00511	Other glycan degradation
0.04	0.02	0.11	0.01	hsa03060	Protein export
0.04	0.01	0.12	0.01	hsa04215	Apoptosis - multiple species
0.04	0.00	0.01	0.01	hsa04310	Wnt signaling pathway
0.04	0.00	0.06	0.01	hsa04622	RIG-I-like receptor signaling pathway
0.04	0.00	0.04	0.01	hsa04630	Jak-STAT signaling pathway
0.04	0.00	0.03	0.01	hsa04660	T cell receptor signaling pathway
0.04	0.00	0.03	0.01	hsa04668	TNF signaling pathway
0.04	0.00	0.10	0.01	hsa04940	Type I diabetes mellitus
0.04	0.00	0.07	0.01	hsa05020	Prion diseases
0.04	0.01	0.13	0.01	hsa05162	Measles
0.03	0.02	0.09	0.01	hsa00040	Pentose and glucuronate interconversions
0.03	0.02	0.12	0.01	hsa00052	Galactose metabolism
0.03	0.01	0.13	0.01	hsa00140	Steroid hormone biosynthesis
0.03	0.02	0.11	0.01	hsa00220	Arginine biosynthesis
0.03	0.00	0.12	0.01	hsa00250	Alanine, aspartate and glutamate metabolism
0.03	0.01	0.13	0.01	hsa00340	Histidine metabolism
0.03	0.00	0.09	0.01	hsa00532	Glycosaminoglycan biosynthesis-chondroitin/dermatan sulfate
0.03	0.00	0.08	0.01	hsa00650	Butanoate metabolism
0.03	0.01	0.06	0.01	hsa00770	Pantothenate and CoA biosynthesis
0.03	0.00	0.17	0.01	hsa00830	Retinol metabolism
0.03	0.00	0.06	0.01	hsa03040	Spliceosome
0.03	0.00	0.02	0.01	hsa04810	Regulation of actin cytoskeleton
0.03	0.00	0.03	0.01	hsa04961	Endocrine and other factor-regulated calcium reabsorption
0.03	0.00	0.10	0.01	hsa04966	Collecting duct acid secretion
0.03	0.00	0.07	0.01	hsa05150	Staphylococcus aureus infection
0.03	0.00	0.06	0.01	hsa05216	Thyroid cancer
0.03	0.00	0.06	0.01	hsa05230	Central carbon metabolism in cancer
0.02	0.00	0.11	0.01	hsa00350	Tyrosine metabolism
0.02	0.00	0.07	0.00	hsa00360	Phenylalanine metabolism
0.02	0.00	0.04	0.00	hsa00500	Starch and sucrose metabolism
0.02	0.00	0.07	0.00	hsa00670	One carbon pool by folate
0.02	0.00	0.04	0.00	hsa00790	Folate biosynthesis
0.02	0.00	0.07	0.00	hsa00860	Porphyrin and chlorophyll metabolism
0.02	0.00	0.04	0.00	hsa03410	Base excision repair
0.02	0.01	0.05	0.00	hsa03430	Mismatch repair
0.02	0.00	0.05	0.01	hsa04066	HIF-1 signaling pathway
0.02	0.01	0.08	0.00	hsa04623	Cytosolic DNA-sensing pathway
0.02	0.00	0.10	0.00	hsa04975	Fat digestion and absorption
0.02	0.00	0.03	0.01	hsa04976	Bile secretion
0.02	0.00	0.05	0.00	hsa05134	Legionellosis
0.02	0.00	0.03	0.00	hsa05310	Asthma
0.02	0.00	0.02	0.00	hsa05322	Systemic lupus erythematosus
0.01	0.00	0.00	0.00	hsa00020	Citrate cycle (TCA cycle)
0.01	0.00	0.04	0.00	hsa00450	Selenocompound metabolism
0.01	0.00	0.01	0.00	hsa00591	Linoleic acid metabolism
0.01	0.00	0.04	0.00	hsa00900	Terpenoid backbone biosynthesis

0.01	0.00	0.02	0.00	hsa00983	Drug metabolism - other enzymes
0.01	0.00	0.00	0.00	hsa03030	DNA replication
0.01	0.00	0.02	0.00	hsa03440	Homologous recombination
0.01	0.00	0.01	0.00	hsa04350	TGF-beta signaling pathway
0.01	0.00	0.00	0.00	hsa04390	Hippo signaling pathway
0.01	0.00	0.02	0.00	hsa05218	Melanoma
0.01	0.00	0.00	0.00	hsa05221	Acute myeloid leukemia
0.00	0.00	0.00	0.00	hsa00190	Oxidative phosphorylation
0.00	0.00	0.01	0.00	hsa00563	Glycosylphosphatidylinositol(GPI)-anchor biosynthesis
0.00	0.00	0.00	0.00	hsa00970	Aminoacyl-tRNA biosynthesis
0.00	0.00	0.00	0.00	hsa03010	Ribosome
0.00	0.00	0.00	0.00	hsa04014	Ras signaling pathway
0.00	0.00	0.00	0.00	hsa04510	Focal adhesion
0.00	0.00	0.00	0.00	hsa05210	Colorectal cancer
0.00	0.00	0.00	0.00	hsa05213	Endometrial cancer
0.00	0.00	0.00	0.00	hsa05215	Prostate cancer
0.00	0.00	0.00	0.00	hsa05220	Chronic myeloid leukemia

Table S3. Agreement measure lambda for benchmarking BRAINEAC against GTEX across 4 brain regions in 287 KEGG pathways.

λ	CI: 2.5%	CI: 97.5%	σ	Ref	Description
0.97	0.99	1.00	0.01	hsa04724	Glutamatergic synapse
0.95	0.97	1.00	0.01	hsa05033	Nicotine addiction
0.94	0.93	1.00	0.01	hsa04360	Axon guidance
0.91	0.93	1.00	0.02	hsa05414	Dilated cardiomyopathy
0.89	0.97	1.00	0.02	hsa04931	Insulin resistance
0.84	0.88	0.99	0.03	hsa04725	Cholinergic synapse
0.84	0.82	0.97	0.02	hsa05032	Morphine addiction
0.82	0.78	0.93	0.02	hsa04720	Long-term potentiation
0.82	0.76	0.89	0.02	hsa05031	Amphetamine addiction
0.81	0.76	0.95	0.02	hsa04912	GnRH signaling pathway
0.79	0.70	0.91	0.03	hsa04261	Adrenergic signaling in cardiomyocytes
0.76	0.75	0.78	0.01	hsa04727	GABAergic synapse
0.75	0.75	0.75	0.00	hsa04080	Neuroactive ligand-receptor interaction
0.75	0.75	0.75	0.00	hsa04723	Retrograde endocannabinoid signaling
0.74	0.75	0.75	0.00	hsa04020	Calcium signaling pathway
0.74	0.73	0.76	0.01	hsa04728	Dopaminergic synapse
0.74	0.76	0.90	0.03	hsa05206	MicroRNAs in cancer
0.73	0.75	0.76	0.01	hsa04015	Rap1 signaling pathway
0.71	0.67	0.95	0.04	hsa04971	Gastric acid secretion
0.71	0.68	0.80	0.02	hsa05034	Alcoholism
0.70	0.61	0.89	0.03	hsa04713	Circadian entrainment
0.69	0.70	0.91	0.04	hsa04144	Endocytosis
0.69	0.59	0.80	0.03	hsa04925	Aldosterone synthesis and secretion
0.69	0.67	0.79	0.02	hsa05030	Cocaine addiction
0.68	0.70	0.83	0.04	hsa04260	Cardiac muscle contraction
0.67	0.64	0.74	0.01	hsa04022	cGMP-PKG signaling pathway
0.66	0.60	0.85	0.05	hsa04742	Taste transduction
0.64	0.49	0.76	0.04	hsa04024	cAMP signaling pathway
0.64	0.55	0.74	0.03	hsa04722	Neurotrophin signaling pathway
0.64	0.71	0.87	0.04	hsa04914	Progesterone-mediated oocyte maturation
0.64	0.62	0.79	0.03	hsa05145	Toxoplasmosis
0.64	0.68	0.85	0.04	hsa05231	Choline metabolism in cancer
0.63	0.55	0.87	0.04	hsa04072	Phospholipase D signaling pathway
0.63	0.74	0.90	0.05	hsa04920	Adipocytokine signaling pathway
0.63	0.61	0.85	0.05	hsa05169	Epstein-Barr virus infection
0.63	0.60	0.87	0.04	hsa05410	Hypertrophic cardiomyopathy (HCM)
0.62	0.56	0.85	0.04	hsa04071	Sphingolipid signaling pathway
0.62	0.71	0.75	0.03	hsa04380	Osteoclast differentiation
0.62	0.70	0.74	0.02	hsa05202	Transcriptional misregulation in cancer
0.61	0.57	0.73	0.03	hsa04010	MAPK signaling pathway
0.61	0.72	0.86	0.05	hsa04146	Peroxisome
0.61	0.53	0.75	0.04	hsa04540	Gap junction
0.61	0.49	0.79	0.05	hsa04911	Insulin secretion
0.61	0.56	0.69	0.03	hsa05203	Viral carcinogenesis
0.60	0.50	0.71	0.03	hsa00230	Purine metabolism
0.60	0.59	0.71	0.02	hsa04142	Lysosome
0.60	0.65	0.79	0.04	hsa04210	Apoptosis
0.60	0.62	0.74	0.02	hsa04670	Leukocyte transendothelial migration
0.59	0.60	0.74	0.03	hsa03015	mRNA surveillance pathway
0.59	0.58	0.74	0.04	hsa04922	Glucagon signaling pathway

0.59	0.59	0.72	0.03	hsa05164	Influenza A
0.58	0.54	0.67	0.02	hsa04062	Chemokine signaling pathway
0.58	0.55	0.73	0.03	hsa04152	AMPK signaling pathway
0.58	0.53	0.71	0.04	hsa04740	Olfactory transduction
0.58	0.68	0.88	0.06	hsa05014	Amyotrophic lateral sclerosis (ALS)
0.57	0.61	0.80	0.04	hsa04114	Oocyte meiosis
0.57	0.55	0.71	0.04	hsa04145	Phagosome
0.56	0.58	0.72	0.03	hsa04150	mTOR signaling pathway
0.56	0.51	0.71	0.03	hsa04611	Platelet activation
0.56	0.51	0.68	0.02	hsa04726	Serotonergic synapse
0.55	0.42	0.81	0.04	hsa04730	Long-term depression
0.54	0.53	0.69	0.04	hsa04070	Phosphatidylinositol signaling system
0.54	0.57	0.79	0.05	hsa04666	Fc gamma R-mediated phagocytosis
0.54	0.52	0.71	0.03	hsa04721	Synaptic vesicle cycle
0.53	0.50	0.63	0.03	hsa05152	Tuberculosis
0.52	0.50	0.59	0.01	hsa04211	Longevity regulating pathway
0.52	0.51	0.62	0.02	hsa05016	Huntington's disease
0.52	0.48	0.69	0.05	hsa05161	Hepatitis B
0.52	0.52	0.60	0.02	hsa05168	Herpes simplex infection
0.51	0.55	0.68	0.04	hsa00270	Cysteine and methionine metabolism
0.51	0.50	0.53	0.01	hsa04213	Longevity regulating pathway - multiple species
0.51	0.48	0.60	0.02	hsa04514	Cell adhesion molecules (CAMs)
0.51	0.60	0.70	0.04	hsa04710	Circadian rhythm
0.51	0.44	0.65	0.04	hsa04915	Estrogen signaling pathway
0.51	0.38	0.59	0.03	hsa04921	Oxytocin signaling pathway
0.50	0.51	0.61	0.02	hsa04919	Thyroid hormone signaling pathway
0.50	0.46	0.66	0.05	hsa04924	Renin secretion
0.49	0.41	0.67	0.03	hsa04970	Salivary secretion
0.48	0.49	0.66	0.04	hsa04664	Fc epsilon RI signaling pathway
0.48	0.42	0.59	0.02	hsa04913	Ovarian steroidogenesis
0.48	0.49	0.50	0.01	hsa05010	Alzheimer's disease
0.47	0.53	0.74	0.05	hsa05140	Leishmaniasis
0.47	0.42	0.64	0.04	hsa05142	Chagas disease (American trypanosomiasis)
0.47	0.49	0.67	0.04	hsa05160	Hepatitis C
0.46	0.47	0.50	0.01	hsa04151	PI3K-Akt signaling pathway
0.46	0.49	0.53	0.02	hsa04550	Signaling pathways regulating pluripotency of stem cells
0.46	0.44	0.53	0.02	hsa05412	Arrhythmogenic right ventricular cardiomyopathy (ARVC)
0.45	0.51	0.67	0.04	hsa00240	Pyrimidine metabolism
0.45	0.44	0.54	0.02	hsa04120	Ubiquitin mediated proteolysis
0.45	0.48	0.62	0.04	hsa04141	Protein processing in endoplasmic reticulum
0.45	0.41	0.62	0.04	hsa05120	Epithelial cell signaling in Helicobacter pylori infection
0.45	0.53	0.67	0.04	hsa05133	Pertussis
0.44	0.47	0.51	0.02	hsa04961	Endocrine and other factor-regulated calcium reabsorption
0.43	0.47	0.62	0.04	hsa00310	Lysine degradation
0.43	0.49	0.69	0.05	hsa00562	Inositol phosphate metabolism
0.43	0.39	0.64	0.04	hsa00564	Glycerophospholipid metabolism
0.43	0.47	0.61	0.04	hsa04650	Natural killer cell mediated cytotoxicity
0.43	0.48	0.50	0.02	hsa04932	Non-alcoholic fatty liver disease (NAFLD)
0.43	0.46	0.50	0.02	hsa05110	Vibrio cholerae infection
0.43	0.33	0.53	0.03	hsa05166	HTLV-I infection
0.43	0.30	0.52	0.03	hsa05205	Proteoglycans in cancer
0.42	0.47	0.75	0.06	hsa00512	Mucin type O-Glycan biosynthesis
0.42	0.40	0.49	0.02	hsa03013	RNA transport
0.42	0.34	0.55	0.04	hsa04012	ErbB signaling pathway

0.42	0.36	0.54	0.03	hsa04930	Type II diabetes mellitus
0.42	0.45	0.50	0.03	hsa05012	Parkinson's disease
0.41	0.41	0.78	0.06	hsa00910	Nitrogen metabolism
0.41	0.35	0.48	0.02	hsa04014	Ras signaling pathway
0.41	0.47	0.50	0.02	hsa04610	Complement and coagulation cascades
0.41	0.45	0.55	0.03	hsa04620	Toll-like receptor signaling pathway
0.41	0.36	0.49	0.02	hsa04923	Regulation of lipolysis in adipocytes
0.41	0.40	0.53	0.03	hsa05323	Rheumatoid arthritis
0.40	0.36	0.50	0.02	hsa04330	Notch signaling pathway
0.40	0.34	0.48	0.02	hsa04750	Inflammatory mediator regulation of TRP channels
0.40	0.39	0.53	0.05	hsa05130	Pathogenic Escherichia coli infection
0.40	0.32	0.53	0.03	hsa05214	Glioma
0.39	0.36	0.58	0.03	hsa00051	Fructose and mannose metabolism
0.39	0.44	0.59	0.04	hsa04622	RIG-I-like receptor signaling pathway
0.39	0.30	0.49	0.03	hsa04933	AGE-RAGE signaling pathway in diabetic complications
0.39	0.30	0.54	0.04	hsa04972	Pancreatic secretion
0.38	0.39	0.58	0.04	hsa04064	NF-kappa B signaling pathway
0.38	0.27	0.47	0.02	hsa04270	Vascular smooth muscle contraction
0.38	0.34	0.49	0.03	hsa04917	Prolactin signaling pathway
0.38	0.39	0.57	0.04	hsa05131	Shigellosis
0.38	0.48	0.70	0.06	hsa05132	Salmonella infection
0.37	0.28	0.49	0.02	hsa02010	ABC transporters
0.37	0.47	0.61	0.05	hsa04662	B cell receptor signaling pathway
0.37	0.36	0.55	0.03	hsa05150	Staphylococcus aureus infection
0.37	0.33	0.46	0.03	hsa05223	Non-small cell lung cancer
0.37	0.29	0.48	0.02	hsa05322	Systemic lupus erythematosus
0.36	0.37	0.50	0.04	hsa00600	Sphingolipid metabolism
0.36	0.36	0.55	0.04	hsa00620	Pyruvate metabolism
0.36	0.37	0.57	0.04	hsa03320	PPAR signaling pathway
0.36	0.27	0.38	0.02	hsa04530	Tight junction
0.36	0.38	0.45	0.03	hsa04668	TNF signaling pathway
0.35	0.36	0.51	0.04	hsa00561	Glycerolipid metabolism
0.35	0.28	0.48	0.03	hsa00604	Glycosphingolipid biosynthesis - ganglio series
0.35	0.35	0.51	0.04	hsa03018	RNA degradation
0.35	0.30	0.43	0.03	hsa04340	Hedgehog signaling pathway
0.35	0.26	0.40	0.02	hsa04510	Focal adhesion
0.35	0.34	0.46	0.03	hsa05219	Bladder cancer
0.34	0.27	0.43	0.02	hsa00510	N-Glycan biosynthesis
0.34	0.44	0.57	0.05	hsa00520	Amino sugar and nucleotide sugar metabolism
0.34	0.30	0.59	0.03	hsa04672	Intestinal immune network for IgA production
0.34	0.25	0.40	0.02	hsa04918	Thyroid hormone synthesis
0.34	0.28	0.42	0.02	hsa05224	Breast cancer
0.33	0.28	0.41	0.02	hsa04066	HIF-1 signaling pathway
0.33	0.27	0.42	0.03	hsa04370	VEGF signaling pathway
0.33	0.26	0.31	0.02	hsa04910	Insulin signaling pathway
0.33	0.30	0.48	0.03	hsa04962	Vasopressin-regulated water reabsorption
0.33	0.29	0.44	0.03	hsa05146	Amoebiasis
0.33	0.35	0.47	0.04	hsa05222	Small cell lung cancer
0.32	0.35	0.46	0.03	hsa00410	beta-Alanine metabolism
0.32	0.25	0.29	0.02	hsa04068	FoxO signaling pathway
0.32	0.25	0.33	0.02	hsa04810	Regulation of actin cytoskeleton
0.32	0.25	0.31	0.02	hsa04916	Melanogenesis
0.32	0.36	0.45	0.04	hsa05212	Pancreatic cancer
0.31	0.32	0.47	0.03	hsa00071	Fatty acid degradation

0.31	0.29	0.58	0.05	hsa00603	Glycosphingolipid biosynthesis - globo series
0.31	0.26	0.48	0.05	hsa00640	Propanoate metabolism
0.31	0.27	0.36	0.02	hsa03050	Proteasome
0.31	0.33	0.44	0.02	hsa03460	Fanconi anemia pathway
0.31	0.26	0.41	0.02	hsa04512	ECM-receptor interaction
0.31	0.25	0.34	0.02	hsa04520	Adherens junction
0.31	0.27	0.43	0.03	hsa04612	Antigen processing and presentation
0.31	0.29	0.47	0.03	hsa04974	Protein digestion and absorption
0.31	0.26	0.41	0.02	hsa05020	Prion diseases
0.31	0.34	0.55	0.05	hsa05144	Malaria
0.31	0.30	0.43	0.03	hsa05217	Basal cell carcinoma
0.30	0.26	0.42	0.03	hsa00630	Glyoxylate and dicarboxylate metabolism
0.30	0.29	0.41	0.04	hsa03040	Spliceosome
0.30	0.25	0.30	0.01	hsa04060	Cytokine-cytokine receptor interaction
0.30	0.29	0.41	0.04	hsa04350	TGF-beta signaling pathway
0.30	0.29	0.49	0.05	hsa04614	Renin-angiotensin system
0.30	0.26	0.32	0.02	hsa04630	Jak-STAT signaling pathway
0.30	0.25	0.29	0.01	hsa04660	T cell receptor signaling pathway
0.30	0.25	0.38	0.01	hsa05100	Bacterial invasion of epithelial cells
0.30	0.29	0.49	0.04	hsa05211	Renal cell carcinoma
0.30	0.28	0.39	0.03	hsa05221	Acute myeloid leukemia
0.29	0.25	0.38	0.02	hsa00260	Glycine, serine and threonine metabolism
0.29	0.31	0.46	0.04	hsa03420	Nucleotide excision repair
0.29	0.34	0.46	0.04	hsa03450	Non-homologous end-joining
0.29	0.31	0.38	0.03	hsa04115	p53 signaling pathway
0.29	0.28	0.45	0.04	hsa04392	Hippo signaling pathway -multiple species
0.29	0.28	0.36	0.03	hsa05215	Prostate cancer
0.29	0.28	0.38	0.03	hsa05218	Melanoma
0.29	0.26	0.34	0.02	hsa05230	Central carbon metabolism in cancer
0.28	0.28	0.57	0.05	hsa00062	Fatty acid elongation
0.28	0.32	0.51	0.04	hsa00531	Glycosaminoglycan degradation
0.28	0.30	0.41	0.04	hsa04130	SNARE interactions in vesicular transport
0.28	0.25	0.26	0.01	hsa04310	Wnt signaling pathway
0.28	0.25	0.34	0.02	hsa04640	Hematopoietic cell lineage
0.28	0.28	0.52	0.05	hsa04973	Carbohydrate digestion and absorption
0.28	0.28	0.43	0.04	hsa04977	Vitamin digestion and absorption
0.28	0.26	0.33	0.01	hsa05213	Endometrial cancer
0.28	0.26	0.32	0.02	hsa05321	Inflammatory bowel disease (IBD)
0.27	0.26	0.36	0.02	hsa00010	Glycolysis / Gluconeogenesis
0.27	0.31	0.38	0.03	hsa00020	Citrate cycle (TCA cycle)
0.27	0.26	0.33	0.03	hsa00100	Steroid biosynthesis
0.27	0.24	0.43	0.04	hsa00280	Valine, leucine and isoleucine degradation
0.27	0.30	0.41	0.04	hsa00565	Ether lipid metabolism
0.27	0.25	0.30	0.01	hsa03410	Base excision repair
0.27	0.25	0.26	0.00	hsa04976	Bile secretion
0.27	0.31	0.39	0.03	hsa04978	Mineral absorption
0.27	0.25	0.31	0.02	hsa05220	Chronic myeloid leukemia
0.27	0.25	0.46	0.03	hsa05320	Autoimmune thyroid disease
0.26	0.29	0.43	0.04	hsa00500	Starch and sucrose metabolism
0.26	0.25	0.31	0.02	hsa00534	Glycosaminoglycan biosynthesis - heparan sulfate / heparin
0.26	0.25	0.25	0.00	hsa04390	Hippo signaling pathway
0.26	0.25	0.25	0.00	hsa05200	Pathways in cancer
0.25	0.25	0.33	0.02	hsa00514	Other types of O-glycan biosynthesis
0.25	0.21	0.41	0.04	hsa00590	Arachidonic acid metabolism

0.25	0.25	0.45	0.03	hsa00760	Nicotinate and nicotinamide metabolism
0.25	0.27	0.39	0.04	hsa03008	Ribosome biogenesis in eukaryotes
0.25	0.25	0.32	0.02	hsa03022	Basal transcription factors
0.25	0.27	0.37	0.04	hsa04621	NOD-like receptor signaling pathway
0.25	0.25	0.26	0.01	hsa04966	Collecting duct acid secretion
0.25	0.25	0.29	0.02	hsa05134	Legionellosis
0.25	0.25	0.26	0.01	hsa05210	Colorectal cancer
0.25	0.24	0.26	0.02	hsa05216	Thyroid cancer
0.25	0.28	0.40	0.04	hsa05340	Primary immunodeficiency
0.24	0.25	0.25	0.01	hsa00480	Glutathione metabolism
0.24	0.22	0.37	0.04	hsa00592	alpha-Linolenic acid metabolism
0.24	0.22	0.39	0.04	hsa04960	Aldosterone-regulated sodium reabsorption
0.24	0.26	0.39	0.04	hsa04964	Proximal tubule bicarbonate reclamation
0.24	0.20	0.45	0.04	hsa05332	Graft-versus-host disease
0.23	0.24	0.25	0.02	hsa00190	Oxidative phosphorylation
0.23	0.24	0.28	0.02	hsa00330	Arginine and proline metabolism
0.23	0.17	0.40	0.04	hsa00533	Glycosaminoglycan biosynthesis - keratan sulfate
0.23	0.24	0.40	0.03	hsa00670	One carbon pool by folate
0.23	0.24	0.32	0.03	hsa04110	Cell cycle
0.22	0.22	0.26	0.02	hsa00380	Tryptophan metabolism
0.22	0.22	0.30	0.04	hsa05162	Measles
0.22	0.19	0.39	0.04	hsa05416	Viral myocarditis
0.21	0.19	0.39	0.04	hsa00511	Other glycan degradation
0.21	0.22	0.41	0.04	hsa00770	Pantothenate and CoA biosynthesis
0.21	0.22	0.31	0.02	hsa00830	Retinol metabolism
0.21	0.23	0.25	0.02	hsa03010	Ribosome
0.21	0.22	0.33	0.04	hsa04215	Apoptosis - multiple species
0.21	0.22	0.40	0.03	hsa05143	African trypanosomiasis
0.21	0.16	0.42	0.04	hsa05330	Allograft rejection
0.20	0.19	0.35	0.03	hsa00350	Tyrosine metabolism
0.20	0.23	0.30	0.02	hsa00430	Taurine and hypotaurine metabolism
0.20	0.18	0.26	0.02	hsa00532	Glycosaminoglycan biosynthesis-chondroitin/dermatan sulfate
0.20	0.18	0.25	0.03	hsa00982	Drug metabolism - cytochrome P450
0.20	0.19	0.31	0.03	hsa04140	Regulation of autophagy
0.20	0.22	0.35	0.03	hsa04744	Phototransduction
0.20	0.12	0.45	0.04	hsa04950	Maturity onset diabetes of the young
0.20	0.17	0.32	0.03	hsa04975	Fat digestion and absorption
0.20	0.17	0.28	0.03	hsa05204	Chemical carcinogenesis
0.19	0.15	0.31	0.04	hsa04940	Type I diabetes mellitus
0.18	0.13	0.32	0.03	hsa00030	Pentose phosphate pathway
0.18	0.15	0.33	0.03	hsa00053	Ascorbate and aldarate metabolism
0.18	0.23	0.27	0.03	hsa00130	Ubiquinone and other terpenoid-quinone biosynthesis
0.18	0.17	0.27	0.03	hsa00140	Steroid hormone biosynthesis
0.18	0.11	0.34	0.04	hsa00601	Glycosphingolipid biosynthesis - lacto and neolacto series
0.18	0.16	0.25	0.03	hsa00980	Metabolism of xenobiotics by cytochrome P450
0.18	0.22	0.33	0.03	hsa05310	Asthma
0.17	0.16	0.31	0.03	hsa00052	Galactose metabolism
0.17	0.16	0.29	0.03	hsa00250	Alanine, aspartate and glutamate metabolism
0.17	0.18	0.34	0.03	hsa01040	Biosynthesis of unsaturated fatty acids
0.17	0.15	0.28	0.04	hsa03020	RNA polymerase
0.17	0.16	0.29	0.03	hsa03060	Protein export
0.17	0.19	0.27	0.03	hsa03430	Mismatch repair
0.17	0.11	0.28	0.03	hsa04320	Dorso-ventral axis formation
0.16	0.15	0.28	0.03	hsa00040	Pentose and glucuronate interconversions

0.16	0.12	0.23	0.02	hsa00563	Glycosylphosphatidylinositol(GPI)-anchor biosynthesis
0.16	0.19	0.26	0.03	hsa00790	Folate biosynthesis
0.16	0.15	0.26	0.03	hsa04623	Cytosolic DNA-sensing pathway
0.15	0.15	0.24	0.03	hsa00120	Primary bile acid biosynthesis
0.15	0.07	0.26	0.03	hsa00591	Linoleic acid metabolism
0.15	0.11	0.24	0.03	hsa00650	Butanoate metabolism
0.15	0.13	0.25	0.02	hsa00860	Porphyrin and chlorophyll metabolism
0.15	0.13	0.24	0.03	hsa00900	Terpenoid backbone biosynthesis
0.15	0.08	0.19	0.02	hsa00970	Aminoacyl-tRNA biosynthesis
0.14	0.14	0.24	0.03	hsa00220	Arginine biosynthesis
0.14	0.08	0.20	0.03	hsa03030	DNA replication
0.13	0.07	0.19	0.03	hsa00061	Fatty acid biosynthesis
0.12	0.06	0.23	0.02	hsa00360	Phenylalanine metabolism
0.12	0.09	0.18	0.02	hsa03440	Homologous recombination
0.11	0.07	0.22	0.02	hsa00340	Histidine metabolism
0.09	0.02	0.13	0.02	hsa00450	Selenocompound metabolism
0.07	0.01	0.10	0.02	hsa00983	Drug metabolism - other enzymes
